# FlowVN Trained on a Single Dataset Enables Rapid Reconstruction of Highly Accelerated 4D Flow MRI Across Multiple Sites

**DOI:** 10.1002/mrm.70317

**Published:** 2026-02-26

**Authors:** Sohaib Ayaz Qazi, Tamara Bianchessi, Federica Viola, Chiara Trenti, Erik Ylipää, Tino Ebbers, Petter Dyverfeldt

**Affiliations:** ^1^ Department of Health, Medicine and Caring Sciences Linköping University Linköping Sweden; ^2^ Center for Medical Image Science and Visualization (CMIV) Linköping University Linköping Sweden; ^3^ Science for Life Laboratory Linköping University Linköping Sweden

**Keywords:** cardiac MRI, deep learning, flow, image reconstruction, turbulent kinetic energy, variational networks

## Abstract

**Purpose:**

The aim of this study is to evaluate a deep variational network, FlowVN, for the reconstruction of heavily undersampled 4D Flow MRI across multiple sites.

**Methods:**

FlowVN was trained on fully sampled 4D Flow MRI datasets of healthy volunteers from one site. The model was tested on retrospective undersampled data (*R* = 6–22) of six normal volunteers from the same site and six from another site, prospectively undersampled data (*R* = 12.4–13.8) of six healthy volunteers from the second site and six patients with aortic stenosis from first Site. Performance was evaluated using nRMSE, relative and angular error, average and maximum velocity, flow rate, volumetric flow, and turbulent kinetic energy (TKE), with a Wilcoxon signed‐rank test to assess the difference from ground truth.

**Results:**

FlowVN showed minimal sensitivity to the number of training datasets and performed well even when trained on a single dataset. FlowVN also demonstrated good generalizability across sites. No significant difference in average and maximum velocity was observed up to *R* = 16. Total TKE was preserved up to *R* = 10 in the normal volunteers, but was well preserved even at higher acceleration factors in the aortic stenosis patients. Flow volumes through the ascending and descending aorta were well preserved for all acceleration factors, although ascending aorta flow volumes for data from one site were significantly different from ground truth for AF > 16.

**Conclusion:**

FlowVN accurately reconstructs highly undersampled 4D Flow MRI from multiple sites using a model trained on a single dataset, maintaining excellent quantitative image quality even at very high acceleration factors.

## 1 Introduction

Time‐resolved, three‐dimensional phase‐contrast MRI with three‐directional velocity encoding (4D Flow MRI) offers comprehensive assessment of cardiovascular hemodynamics [1]. Applications of 4D Flow MRI in the cardiovascular, neurovascular and hepatic portal system have led to several novel insights into the role of blood flow [2, 3, 4, 5, 6]. Clinically, 4D Flow is particularly useful in settings such as complex congenital and structural heart diseases where the opportunity to visualize complex flows and retrospectively quantify flow volumes at multiple locations offers several advantages compared to conventional 2D through‐plane flow measurements [7, 8, 9, 10]. In addition to velocity mapping, which uses the effects of moving tissue on the phase of the complex‐valued phase‐contrast MRI signal, 4D Flow MRI turbulence mapping permits estimation of turbulent kinetic energy (TKE) by exploiting the effects of turbulent flow on the magnitude of the phase‐contrast MR signal [11, 12].

An important limitation of 4D Flow MRI is its inherently long acquisition time, which can lead to patient discomfort and limited applicability in clinical diagnostics. Conventional parallel imaging is the most widely used acceleration technique for 4D Flow but is limited to relatively modest acceleration factors [13, 14]. Recently, compressed sensing (CS) has become increasingly available and permits high acceleration factors [15], with good 4D Flow data quality reported for an acceleration factor of 10 [16, 17]. However, CS is hampered by limitations which include (i) the need for data‐specific sparsifying transforms because of different MR imaging modalities, (ii) a risk for unnatural appearance of the reconstructed images, (iii) the need to empirically tune regularization parameters for each type of data, and (iv) the use of computationally expensive and time‐consuming iterative algorithms [18, 19].

Deep learning has the potential to offer very short reconstruction times for high acceleration factors and overcome limitations of conventional reconstruction methods such as CS and parallel imaging. Several networks have demonstrated encouraging results for phase‐contrast MRI [20, 21, 22, 23, 24, 25, 26, 27, 28]. For example, Vishnevskiy et al. presented a deep variational neural network termed FlowVN and achieved reconstructions of highly undersampled 4D flow MRI data in less than a minute [22]. Based on the work of Hammernik et al. [29], FlowVN models the CS reconstruction using an unrolled gradient descent framework. The network learns all the regularization parameters during training and mimics the iterative nature of CS. In a recent study, Li et al. proposed a pretraining followed by ADMM finetuning optimization algorithm for 4D Flow MRI reconstruction and compared it with FlowVN [30], where FlowVN showed comparable performance for aortic velocity metrics. Recently, FlowMRI‐Net was proposed as a generalizable self‐supervised deep learning framework for reconstructing highly undersampled 4D Flow MRI across multiple vendors and anatomical regions, demonstrating improved velocity accuracy compared to CS‐LLR methods [31]. This reflects a growing interest in evaluating the generalizability of reconstruction networks. However, FlowVN has only been tested at one site. Additional applications of FlowVN and reconstruction of data from multiple sites can provide further insight into the applicability of this network. Moreover, the capability of FlowVN to accurately reconstruct not only the phase but also the magnitude of the phase‐contrast MR signal for velocity and TKE mapping, respectively, has not been explored.

The aim of this study was to (1) implement and train FlowVN at a different site, for the reconstruction of both velocity and TKE images and (2) explore the sensitivity of FlowVN to the number of training datasets as well as its generalizability on data from different sites.

## 2 Methods

### 2.1 Data

Fully‐sampled whole‐heart cartesian 4D flow MRI data were acquired at our institution (referred to as Site A) using a Philips 1.5 T Achieva scanner (Philips Healthcare, Best, The Netherlands) with software version 5.7.1, in 16 healthy volunteers during free breathing. The volunteers were aged between 26 and 41 years and self‐identified as 6 male, 9 female, and 1 non‐binary. Parameter settings included image size = 112 × 112, slices varying from 52 to 79, repetition time = 4.82 ms, echo time = 3.1 ms, *k*‐space segmentation factor 3, flip angle = 15°, velocity encoding limit (VENC) = 150 cm/s, acquired spatial resolution = 2.5 × 2.5 × 2.5 mm^3^ and acquired temporal resolution = 57.8 ms. Electrocardiogram gating was applied to synchronize data acquisition with the cardiac cycle and weighted navigator gating with a 5 mm gating window for the 25% central *k*‐space and a 15 mm window for the outer 75% of *k*‐space was used to acquire data at end expiration and thereby reduce breathing artifacts. Overall scan time, for the fully sampled acquisitions, was 30–40 min including navigator gating.

The healthy volunteer data was reconstructed to the acquired number of cardiac phases (ranging from 9 to 17), which depended on heart rate (“duration of cardiac cycle”/“acquired temporal resolution”). To meet the requirements of the network for training and make data size consistent, the number of cardiac phases in the training datasets were increased by duplicating the entire cardiac sequence and appending it to the end of the original sequence.

Raw data was exported from the scanner and read using ReconFrame (GyroTools LLC, Zurich, Switzerland) in MATLAB R2023a (MathWorks Inc., Natick, Massachusetts, USA). The fully sampled data was acquired with 22–25 coils and was compressed to 5 coils to reduce the data size, using the ESPIRiT‐based coil compression method in the BART Toolbox [32, 33]. Sensitivity maps were generated using the ESPIRiT method with BART Toolbox [32, 34] and are used to enforce data consistency in FlowVN training and testing. The volunteer data acquired from Site A was randomly split into training and validation (*n* = 10) and test data (*n* = 6). The test data from Site A was retrospectively undersampled using Cartesian golden angle sampling, which results in variable density random undersampling [35], for acceleration factors ranging from 6 to 22. This undersampling technique uses temporal *k*‐space filtering which exploits the redundancy in the data across time to recover missing spatial information in one time frame using the information from other time frames [35].

Additionally, in order to test the network in settings of elevated TKE, 4D Flow MRI data were acquired in six patients (age 48–69 years, three self‐identified as male and three as female) with aortic stenosis at Site A, with the same scanner and similar acquisition parameters as for the healthy volunteers. These patient data were acquired with a compressed SENSE undersampling factor of eight and reconstructed to full data using the BART PICS Tool [32]. The reconstructed data were treated as ‘reference’ data and were subsequently undersampled in the same way as the healthy volunteer data.

To test the network on data from a different site, 12 publicly available 4D Flow datasets from Vishnevskiy et al. [22] (ETH, Zürich, Switzerland, https://codeocean.com/capsule/0115983/tree) were used as an additional test set, referred to as Site B in the remainder of the article. These datasets were acquired on a 3 T Philips Ingenia System with VENC = 150 cm/s. Six of the 12 publicly available 4D Flow datasets were also undersampled using the same Cartesian golden angle sampling pattern [22] and undersampling factors as the healthy volunteer test data from Site A, and the other six had been acquired (prospective undersampling) with acceleration factors of ˜13 using the same Cartesian golden angle sampling pattern as described above.

### 2.2 Network and Training

We adapted FlowVN for TensorFlow 2.6.0. Modifications compared to the network published by Vishnevskiy et al. [22] include a revised data pipeline for training and validation with efficient batch handling. The network uses 10 unrolled layers, where each layer corresponds to an iteration in CS reconstruction. The unroll method employed was gradient descent momentum. Each 3D filter bank contains 8 convolution tunable filters and the convolutional layers of size [5, 5, 5] across the 10 unrolled layers. Linear interpolation was used with 91 knots, and the initial learning rate was set to $1.0e^{-3}$ for ADAM Optimizer $\left(\beta_{1}=0.85,\beta_{2}=0.98\right)$. The linear activation function operated within a fixed range of [−3.0, 3.0], while the data activation range was set between [−7, 7]. Variable‐density random undersampling was applied using a gamma parameter ranging from 0.04 to 0.35, which controlled the sampling probability across *k*‐space (with a denser *k*‐space center), resulting in continuously varying effective acceleration factors between approximately 6 and 22. All the learnable parameters were initialized from scratch for each training model, without any pretrained weights from previous studies. All five coils were included during batch selection to enhance data coverage and ensure the network was exposed to the complete coil information during training. The network uses coil sensitivity maps and cartesian golden angle sampling to enforce data consistency during training and testing. The number of iterations per training run was empirically set to 12 000 (based on tests on Site A data), while the rest of the training parameters were the same as those reported by Vishnevskiy et al. [22]. The updated network, a trained model and a test dataset from Site A are publicly available at https://gitlab.liu.se/cim_public/4dflowvn‐cross‐site‐rapid‐cmr.

To assess the sensitivity of the network to the amount and choice of training data, the network was trained 15 times separately (training runs), with a different number of training datasets ranging from 1 to 5 in each single training run. For each training run, the training datasets were selected from *n* = 10 pool using a round‐robin approach. For each training run, 5 datasets not involved in the training, selected randomly, were used for validation. Trainings 1–15 used *n* = [1 1 1 2 2 2 3 3 3 4 4 4 5 5 5] datasets. During each iteration of one training run, a random batch of size $n_{\mathrm{xs}}\times n_{y}\times n_{z}\times n_{\mathrm{ts}}$ was selected from the training set. For a specific batch, the size of x‐dimension (xs) was 14, as well as the number of selected cardiac phases (ts). The size of xs‐dimension and cardiac phases, ts, was determined through empirical testing, involving multiple training runs and result comparisons on Site A data to optimize performance. Network training was done on a computer equipped with an NVIDIA GeForce RTX 4090Ti GPU. The training time for each training run was 21 h.

### 2.3 Testing

**FIGURE 1:**
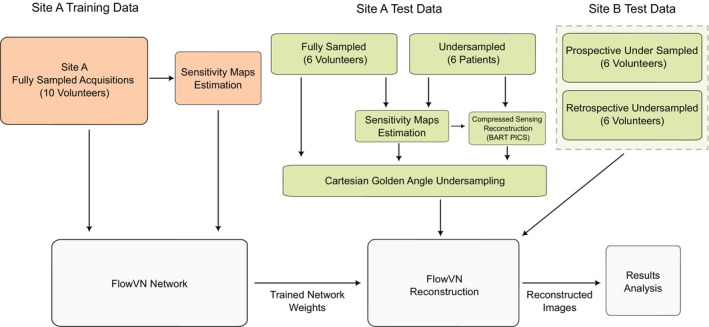
Flow diagram of the methodological approach used to assess the performance of FlowVN.

The trained models were tested on a holdout dataset comprising six of the 16 fully sampled healthy volunteer datasets acquired at Site A, which were not used during training and validation samples. We also tested the trained models on the 12 publicly available 4D Flow MRI datasets from Site B (six retrospectively undersampled and six prospectively undersampled) and on the six aortic stenosis patient datasets acquired at Site A. All tests were done for undersampling factors ranging from 6 to 22 for retrospectively undersampled data (both Site A and B), while the prospectively undersampled data had acceleration factors of 12.4–13.8. The reconstruction time for one full 4D Flow acquisition was less than 2 min for each undersampling factor. The acquisition and processing of training and test data are summarized in Figure 1.

### 2.4 Image Quality Evaluation

The thoracic aorta was segmented using a time‐resolved deep learning‐based automated method [36], and matched to the coverage of the segmentation of the data from Site B (see Figure S1). Three cross‐sectional planes were positioned manually in the ascending and descending aorta (see Figure S1). Segmentation and plane positioning was done once in the ground truth data and applied to the FlowVN reconstructed data. Image quality was assessed quantitatively based on the relative error of velocity magnitude in the thoracic aorta, the angular error (angular dissimilarity between velocity vectors) in the thoracic aorta, and the normalized root mean square error (nRMSE) of signal intensity in the whole magnitude image and are described as:

$$\text{RelErr}\;\left(a,a^{*}\right)=\frac{{\left\|a-a^{*}\right\|}_{2}}{{\left\|a^{*}\right\|}_{2}}$$

$$\text{AngErr}\;\left(u,v\right)=\arccos\left(\frac{\left\langle u,v\right\rangle }{{\left\|u\right\|}_{2}{\left\|v\right\|}_{2}}\right)$$

$$\text{nRMSE}\;\left(a,a^{*}\right)=\sqrt{\sum_{i}^{N}\frac{{\left(a_{i}-a_{i}^{*}\right)}^{2}}{N\max_{j}{\left(a_{j}^{*}\right)}^{2}}}$$

All metrics were computed voxel‐wise and subsequently averaged across all voxels to obtain a single representative value per volunteer and acceleration factor. Additionally, the average and maximum (99.99th percentile) flow velocity and the total, spatially integrated TKE [37] in the thoracic aorta were computed at each cardiac phase. To reduce noise, a 3D median filter (3 × 3 × 3 kernel size) was applied to the TKE data before analysis. Further, flow volumes (FV) through a plane in the mid‐ascending aorta (AAo) and a plane in the descending aorta (DAo) were computed (using in‐house tool) by taking the time integral of flow rate over the cardiac cycle. A second‐order correction was used for background phase offsets before calculating flow in the aorta [38]. All the evaluation metrics were computed for both ground truth and FlowVN reconstructed data.

The uncertainty in performance metrics due to variations in the size and configuration of the training set, was evaluated by calculating the coefficient of variation, $\mathrm{COV}=\frac{\text{Standard Deviation}\;\left(\mathrm{SD}\right)}{\text{MEAN}}\times 100$, for all the performance metrics. The coefficient of variation represents the percentage variability of results over different training runs, relative to the mean. Given the small sample size, a Wilcoxon signed‐rank test was used to evaluate differences between reconstructed results and ground truth for the healthy volunteers and aortic stenosis patients; a *p*‐value < 0.05 was considered statistically significant. In addition, a relative mean difference of 10% was considered clinically acceptable for average and maximum velocities as well as for flow rates.

### 2.5 Ethics Approval and Consent

The study was approved by the Swedish Ethical Review Authority. Written informed consent was obtained from each participant.

## 3 Results

### 3.1 Performance of FlowVN With Varying Training Dataset Sizes

**FIGURE 2:**
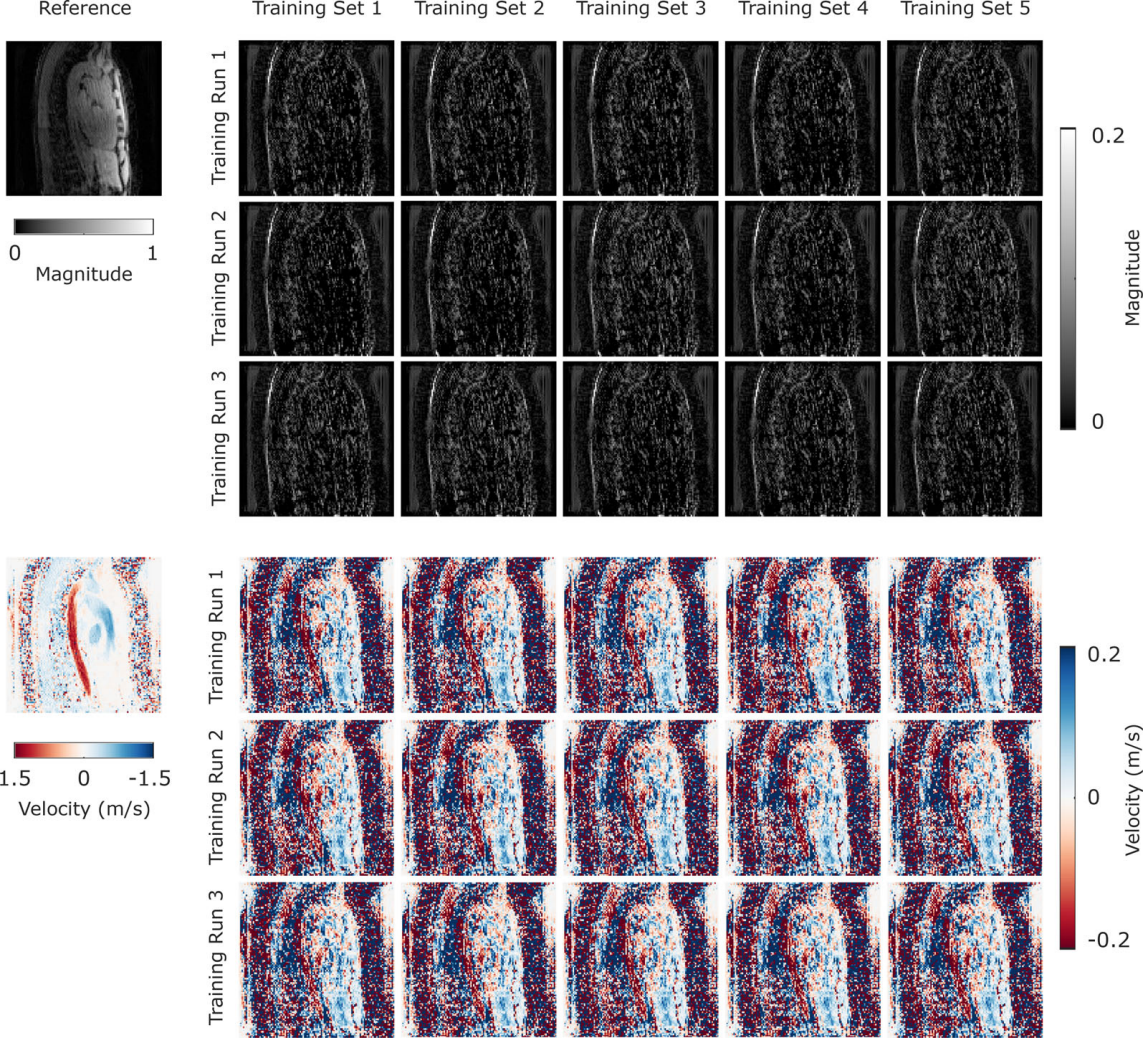
Examples of 4D Flow MRI reference magnitude and velocity images (first column) for data from Site A. For each row, columns 2–6 show the differences between the reference and images reconstructed with models trained on 1–5 (Training Set 1–5 respectively) datasets. Note that there are no apparent systematic differences due to the size of the training set.

The nRMSE, the relative error, and the angular error were similar for 4D Flow MR images reconstructed with FlowVN models trained on 1, 2, 3, 4 or 5 4D Flow MRI datasets (Figure S2 and Figure 2). Figure S2 box plots (grouped by training type and AF) show similar medians across models and training‐set sizes. The first column in Figure 2 shows magnitude and velocity images of fully sampled reference data, while columns 2–6 show the difference images between reconstructions from models trained on 1–5 datasets and the reference in column 1. Coefficients of variation (CoV) of several image quality metrics and hemodynamic parameters for all 15 training models for Site A and Site B are shown in Figure S3. The CoV shows a similar trend in results from both sites across different training models. The underlying mean and standard deviation values can be found in Tables S1–S4. In general, higher variation is observed at higher acceleration factors for most of the performance metrics, and there is a similar trend in the results from Site A and B across different training models. For nRMSE, angular error and relative error, we observed a decreased variation at higher acceleration factors. As the number of datasets used for training did not affect the reconstruction results, the remainder of the results presented in this paper represent reconstructions with a model trained on a single dataset, selected randomly (training run 2 with training set 1 in Figure 2).

### 3.2 Retrospective Evaluation

#### 3.2.1 Magnitude and Velocity Image Quality

**FIGURE 3:**
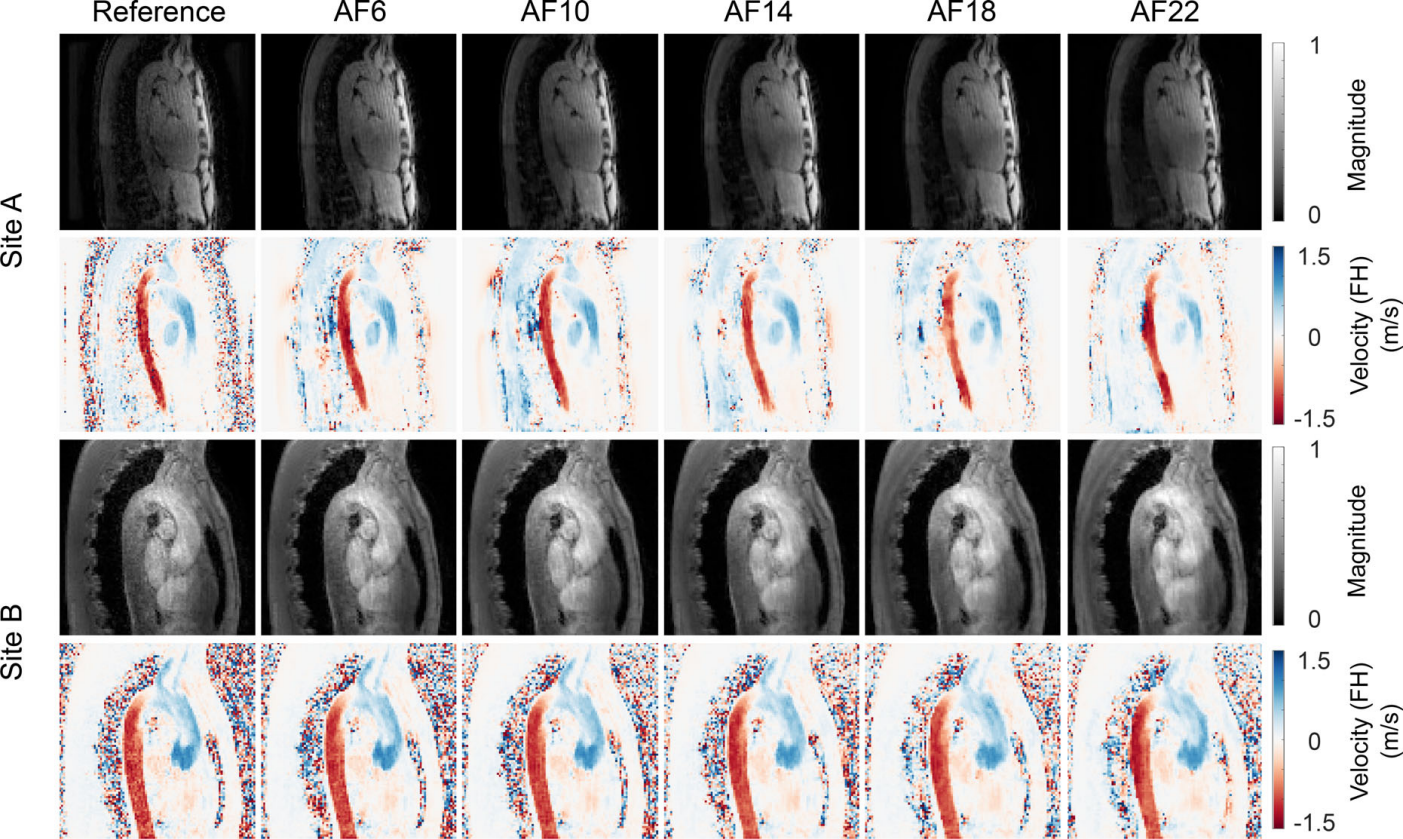
Example of ground truth and FlowVN‐reconstructed magnitude and foot‐to‐head (FH) velocity images for acceleration factors 6, 10, 14, 18 and 22 for one dataset from each Site A and one from Site B. The FlowVN model was trained with a single dataset from Site A.

Representative magnitude and feet‐to‐head velocity images for a subset of evaluated acceleration factors for one dataset from Site A and one (retrospectively undersampled) from Site B are shown in Figure 3. nRMSE, relative error, and angular error for all the evaluated acceleration factors for all six volunteers from Site A and all six volunteers from Site B are presented in Table S5. The data quality is well preserved at high acceleration factors, but there is a trend towards increasing smoothness with increasing acceleration factors.

#### 3.2.2 Velocity in the Thoracic Aorta

**FIGURE 4:**
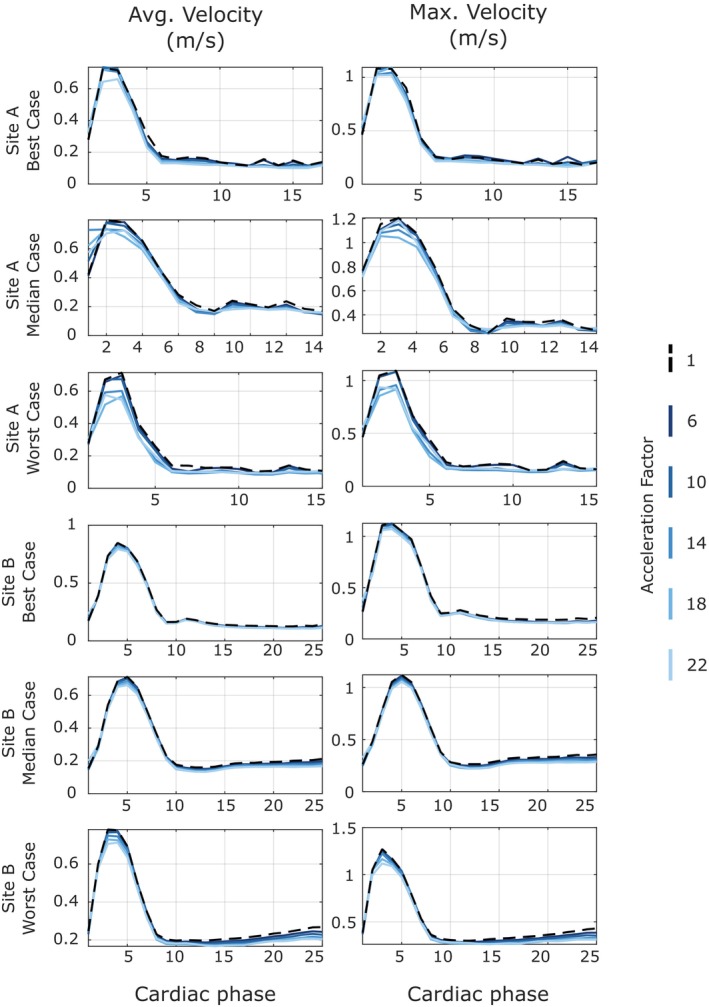
Plots of average and maximum velocity in the thoracic aorta for three representative cases (best, median, worst) from Site A and Site B volunteer datasets at various acceleration factors, reconstructed with a model obtained by training with a single Site A dataset.

Plots of average and maximum velocity in the thoracic aorta over the cardiac cycle for different acceleration factors for the best, median and worst cases from Site A and Site B datasets reconstructed with the model obtained by training with a single dataset from Site A are shown in Figure 4. The representative cases from Site A and Site B were selected based on the mean average and maximum velocity across all acceleration factors. Visually, there is an overall excellent agreement between the accelerated data and ground truth for average and maximum velocity curves across the entire range of acceleration factors. Mean and standard deviation values of maximum and average velocity at systole, along with *p*‐values on the difference between accelerated and ground truth data (for all six volunteers from Site A and Site B), are shown in Table 1. Quantitatively, the Wilcoxon signed‐rank test showed no significant difference for maximum and average velocity across most of the acceleration factors. Significant difference was observed only for AF10 for Site A with a *p*‐value of 0.031 for both average and maximum velocity (Table 1).

**TABLE 1:** Differences in maximum and average velocity at systole, total TKE at systole, average total TKE in whole cardiac cycle, and flow volumes at different acceleration factors (AF) compared to ground truth in the volunteers from Site A and B.

Differences values	Site	AF 6	AF 8	AF 10	AF 12	AF 14	AF 16	AF 18	AF 20	AF 22
Average velocity (m/s)	A	−0.01 ± 0.04 (0.313)	−0.01 ± 0.05 (0.219)	0.0 ± 0.03 (**0.031**)	0.01 ± 0.05 (0.062)	0.04 ± 0.05 (0.156)	0.05 ± 0.06 (0.156)	0.06 ± 0.04 (0.219)	0.03 ± 0.04 (0.156)	0.09 ± 0.043 (0.437)
	B	0.003 ± 0.021 (0.562)	0.004 ± 0.02 (1.000)	0.008 ± 0.02 (1.000)	0.012 ± 0.02 (0.312)	0.02 ± 0.02 (1.000)	0.022 ± 0.02 (1.000)	0.03 ± 0.02 (0.687)	0.04 ± 0.02 (0.687)	0.06 ± 0.023 (0.844)
Max velocity (m/s)	A	−0.02 ± 0.06 (0.156)	0.01 ± 0.07 (0.156)	0.0 ± 0.06 (**0.031**)	0.03 ± 0.07 (0.062)	0.06 ± 0.07 (0.062)	0.07 ± 0.09 (0.094)	0.08 ± 0.07 (0.156)	0.02 ± 0.07 (0.094)	0.08 ± 0.06 (0.062)
	B	0.037 ± 0.06 (0.688)	0.04 ± 0.068 (0.437)	0.049 ± 0.07 (0.437)	0.05 ± 0.07 (1.000)	0.065 ± 0.07 (0.219)	0.07 ± 0.07 (0.688)	0.08 ± 0.06 (0.844)	0.09 ± 0.07 (0.562)	0.11 ± 0.06 (0.562)
Total TKE (mJ)	A	−0.79 ± 0.67 (**0.031**)	−0.88 ± 0.57 (**0.031**)	−0.83 ± 0.56 (**0.031**)	−1.05 ± 0.38 (**0.031**)	−1.26 ± 1.09 (0.062)	−1.37 ± 1.51 (0.093)	−1.25 ± 0.9 (0.062)	−1.19 ± 1.41 (0.156)	−2.64 ± 2.33 (0.094)
	B	−0.23 ± 0.1 (**0.031**)	−0.4 ± 0.21 (**0.031**)	−0.58 ± 0.21 (**0.031**)	−1.13 ± 0.69 (**0.031**)	−1.73 ± 0.7 (**0.031**)	−1.94 ± 0.67 (**0.031**)	−2.8 ± 1.21 (**0.031**)	−3.23 ± 1.73 (**0.031**)	−3.62 ± 1.62 (**0.031**)
Average total TKE (mJ)	A	1.84 ± 0.92 (0.687)	1.9 ± 1.03 (1.000)	1.92 ± 1.00 (1.000)	2.06 ± 1.04 (0.562)	2.47 ± 1.53 (**0.031**)	2.52 ± 1.44 (**0.031**)	2.58 ± 1.51 (**0.031**)	2.57 ± 1.5 (**0.031**)	2.77 ± 1.56 (**0.031**)
	B	−0.25 ± 0.67 (0.094)	−0.21 ± 0.66 (0.062)	−0.15 ± 0.59 (0.062)	−0.07 ± 0.59 (0.062)	0.05 ± 0.54 (0.062)	0.12 ± 0.51 (0.062)	0.29 ± 0.51 (0.062)	0.39 ± 0.5 (0.062)	0.47 ± 0.53 (0.062)
Flow volume AAo (mL)	A	1.23 ± 1.29 (0.094)	1.1 ± 3.46 (0.562)	1.7 ± 1.85 (0.156)	2.83 ± 3.22 (0.156)	3.04 ± 2.79 (0.063)	4.94 ± 5.8 (0.156)	6.12 ± 3.91 (**0.031**)	6.18 ± 3.64 (**0.031**)	6.59 ± 5.91 (**0.031**)
	B	−1.15 ± 5.4 (1.000)	−1.33 ± 5.86 (0.844)	−2.17 ± 5.32 (0.562)	−2.52 ± 5.14 (0.437)	−0.2 ± 5.57 (0.844)	−2.35 ± 4.83 (0.437)	0.04 ± 6.43 (1.000)	−0.06 ± 5.82 (1.000)	0.01 ± 5.77 (0.562)
Flow volume DAo (mL)	A	−0.76 ± 1.45 (0.312)	−0.71 ± 2.82 (0.562)	−1.31 ± 4.52 (0.562)	−0.02 ± 4.1 (0.844)	0.69 ± 6.38 (0.844)	1.15 ± 4.07 (0.562)	2.55 ± 9.57 (0.844)	0.9 ± 8.08 (0.844)	2.41 ± 8.01 (0.687)
	B	1.68 ± 4.68 (0.437)	1.5 ± 4.52 (0.437)	1.48 ± 4.71 (0.437)	0.98 ± 4.13 (0.437)	1.94 ± 3.91 (0.219)	1.97 ± 4.29 (0.219)	2.4 ± 4.6 (0.219)	2.43 ± 5.99 (0.312)	2.31 ± 4.46 (0.437)

*Note*: Data were evaluated in the ascending and descending aorta and are presented as mean ± standard deviation (*p*‐value, Wilcoxon signed‐rank test). Bold values indicate statistically significant differences compared to grouth truth.

#### 3.2.3 TKE in the Thoracic Aorta

**FIGURE 5:**
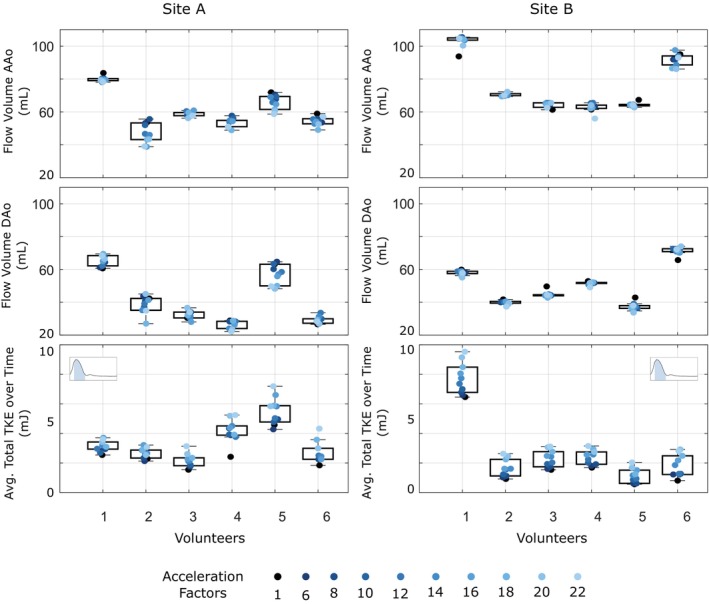
Flow volume (mL) through a plane in ascending aorta (AAo) and descending aorta (DAo) and total TKE averaged over the 10%–35% of the cardiac cycle (highlighted blue in the subfigure) for all six Site A and all six Site B volunteers at various acceleration factors, reconstructed with a model obtained by training with a single Site A dataset.

Plots of total TKE in the thoracic aorta over the cardiac cycle for different acceleration factors for the best, median and worst cases from Site A and Site B datasets reconstructed with the model obtained by training with a single dataset from Site A are shown in Figure S4. There is a fair visual agreement with ground truth up to acceleration factors of around 10, but significant differences in peak total TKE for most acceleration factors, in the normal volunteers with overall low TKE (Figure 5 and Table 1). Least agreement is seen at low TKE values, which are highly susceptible to noise and reconstruction artifacts. Better agreement between reconstructed and reference total TKE is observed at higher TKE levels (> 10 mJ), particularly up to acceleration factor 12 (Figure S4: B and F). The trend is further supported by the Table S6, showing differences in time‐averaged total TKE between ground truth and reconstructed data, which are < 0.5 mJ, for acceleration factors uptill 12 for volunteer 5 from Site A, while average total TKE remains < 2 mJ for all other subjects.

**FIGURE 6:**
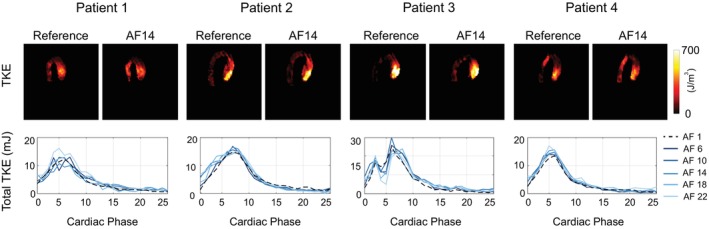
Example of reference and FlowVN‐reconstructed TKE MIPs for acceleration factor 14 and plots of total TKE in thoracic aorta for four patients with aortic stenosis. AF = acceleration factor.

Figure 6 shows reference and FlowVN‐reconstructed maximum intensity projection images (MIPs) of systolic TKE at acceleration factor 14, along with plots of total TKE in the thoracic aorta over the cardiac cycle for the four patients with aortic stenosis. The reconstruction was performed for various acceleration factors, with a model obtained by training with a single dataset. Mean and standard deviation values of total TKE at systole, along with *p*‐values for the difference between accelerated and ground truth data, are shown in Table 2 for all six aortic stenosis patients. Wilcoxon signed‐rank test showed no significant difference in total TKE values across most acceleration factors (AF = 16 showed a significant difference). In contrast to the results for low TKE volunteers, plots of total TKE in the thoracic aorta in patients with aortic stenosis align well with the reference for all acceleration factors (Figure 6).

**TABLE 2:** Differences between total TKE at systole at different acceleration factors (AF) compared to ground truth in aortic stenosis patients.

Difference values	AF 6	AF 8	AF 10	AF 12	AF 14	AF 16	AF 18	AF 20	AF 22
Total TKE (mJ)	−1.20 ± 2.58 (0.312)	−1.58 ± 4.70 (0.844)	−2.35 ± 3.20 (0.156)	−2.83 ± 3.88 (0.094)	−0.84 ± 4.89 (0.844)	−2.23 ± 1.85 **(0.031)**	−0.07 ± 2.61 (1.000)	−2.78 ± 5.08 (0.31)	−4.69 ± 7.65 (0.094)

*Note*: Data are presented as mean ± standard deviation of the difference (*p*‐value, Wilcoxon signed‐rank test). Bold values indicate statistically significant differences compared with the ground truth.

#### 3.2.4 Flow Through Planes in the Ascending and Descending Aorta

**FIGURE 7:**
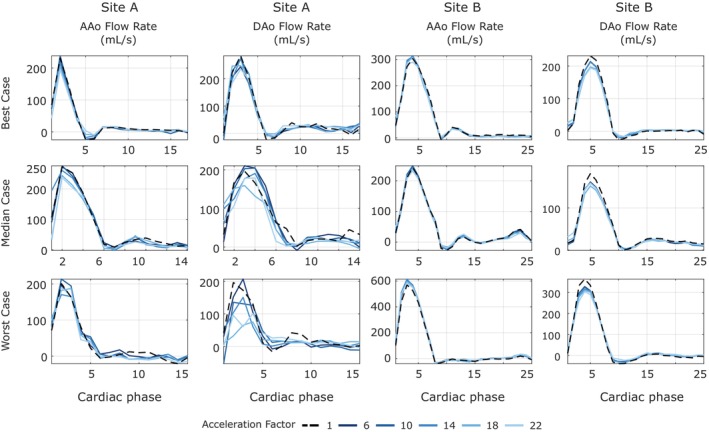
Plots of flow rate in ascending aorta (AAo) and descending aorta (DAo) for the best, median, and worst Site A and Site B datasets at various acceleration factors, reconstructed with a model obtained by training with a single Site A dataset.

Flow rates at one plane in the ascending (AAo) and one plane in the descending (DAo) aorta for a subset of acceleration factors for the best, median and worst cases from Site A and Site B, reconstructed with the model obtained by training with a single dataset from Site A, are shown in Figure 7. In the majority of cases, there is a good agreement between the ground truth and different acceleration factors, with less variation between acceleration factors in the Site B data than the Site A data. The box plot in Figure 5 shows variation of flow volumes for all acceleration factors for all six Site A and all six Site B datasets. We observe less variation in flow volume values across acceleration factors for Site B compared to Site A. In contrast, Site A data shows a higher variation in flow volume values with increasing acceleration factors, and this trend is similar for most of the volunteers (See Figure 5). The quantitative evaluations support these observations (see Table 1). For flow volume in the AAo, Site A data exhibited significant differences and a higher variability at higher acceleration factors, whereas Site B data agreed more closely with the ground truth. No significant difference was observed for the flow volume in DAo for the data from both sites.

### 3.3 Prospective Evaluation

**FIGURE 8:**
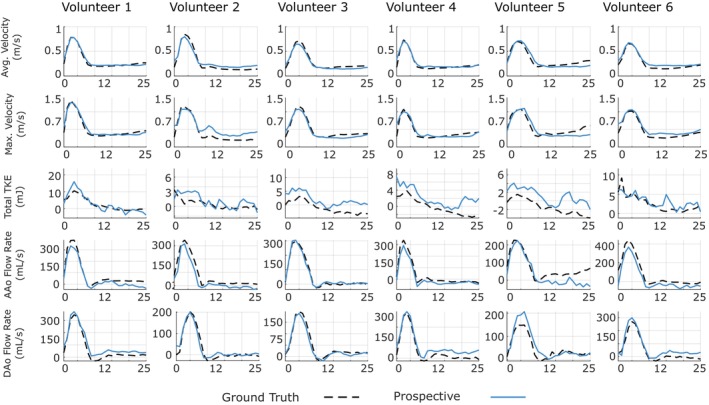
Plots of average and maximum velocity, total TKE in the thoracic aorta, and flow rate in ascending aorta (AAo) and descending aorta (DAo) of ground truth (fully sampled) and six prospectively undersampled datasets from Site B, with various acceleration factors ranging from 12.4 to 13.8. The reconstruction was performed with a model obtained by training with a single site A dataset.

Results on the performance of FlowVN for prospectively undersampled data are shown in Figure S5 and Figure 8. Magnitude and feet‐to‐head velocity images for reconstructions of prospectively undersampled data for six volunteers (acceleration factors range from 12.4 to 13.8) from Site B are shown in Figure S5.

Plots of average and maximum velocity in the thoracic aorta over the cardiac cycle for the prospectively undersampled acquisitions are shown in Figure 8. Overall, there is good agreement between ground truth (fully sampled) and the average and maximum velocity for different acceleration factors, although deviations from the ground truth are observed during diastole for volunteers 5 and 6. Wilcoxon signed‐rank test showed no significant difference in maximum velocity (0.03 ± 0.07 m/s, *p* = 0.313) and average velocity (0.001 ± 0.03 m/s, *p* = 0.844).

Plots of total TKE in thoracic aorta for prospectively undersampled data are presented in Figure 8. The trend is similar to that seen for retrospectively undersampled data. On average, the accelerated reconstruction overestimated total TKE values (−2.54 ± 0.72 mJ) compared to ground truth, with a statistically significant difference *p* = 0.0312.

Flow rate curves for the prospectively undersampled volunteers are presented in Figure 8. The overall performance closely matches that of the retrospectively undersampled data, with good agreement across all volunteers. Similar to retrospective results, the reconstructions for accelerated data showed no significant difference in DAo peak flow rate across volunteers (−21.2 ± 22.7 mL/s, *p* = 0.09), and the values in AAo peak flow rate were also maintained with no significant difference from ground truth (34.8 ± 34.8 mL/s, *p* = 0.16).

## 4 Discussion

4D Flow MRI is on the verge of widespread clinical application but is still limited by long scan and reconstruction times. Deep learning methods are promising for rapid reconstruction of highly undersampled data but require careful evaluation prior to widespread utility in clinical and research studies. Here, we thoroughly evaluated a variational neural network termed FlowVN and found that it permits rapid and accurate reconstructions of highly undersampled 4D Flow MRI data from multiple sites when trained only on a single 4D Flow MRI dataset, while preserving the image quality for accurate measurement of velocity, flow and TKE in thoracic aorta.

FlowVN models obtained by training on only one 4D Flow MRI dataset performed on par with models obtained by training on five datasets. Previously, Vishnevskiy et al. [22] used 11 and Li et al. [30] used 9 datasets for FlowVN training but did not report results on the impact of the size of the training set. Unlike conventional deep neural networks with millions of free parameters (e.g., 60 million for AlexNet) which require large and diverse datasets, FlowVN is based on principles of CS and has an architecture with relatively few learnable parameters (∼71 000) and inherently more structure which constrains what it learns. Compared to the version of FlowVN made available by Vishnevskiy et al. [22], we modified the batch selection process to ensure that all coils were included during training. These modifications may support more efficient learning. FlowVN trains to map from *k*‐space to image domain for a variety of undersampling factors, for each patch (12 000 patches for one training model). As FlowVN is designed to learn this well‐defined operation, it does not require training on data from various 4D Flow datasets to generalize effectively. The CS‐based architecture of FlowVN and the patch‐based learning appear to enable efficient training even with limited data.

Our comprehensive evaluation of the performance of FlowVN for several image quality and hemodynamic parameters demonstrates good reconstruction capabilities for 4D Flow MRI data. In addition to the findings presented by Vishnevskiy et al. [22], peak and average velocities in the aorta are maintained even at very high acceleration factors >20. The mean difference also remained below 10%, which may be considered clinically acceptable for thoracic velocity measurements. In addition to peak and average velocities, we also obtained highly encouraging results on flow rate curves, both for retrospectively and prospectively undersampled data. Similar to the results on maximum and average velocity, flow rate and flow volumes are well preserved in data reconstructed with FlowVN up till acceleration factor 16. Although for flow volume we observed significant differences at higher acceleration factors, the difference remained within 10% of the ground truth values. Good performance of FlowVN for highly undersampled 4D Flow MRI was also recently reported by Li et al. who compared a novel unsupervised deep learning reconstruction method against FlowVN for acceleration factors up to 16 [30]. FlowVN performed similarly as the method proposed by Zhonsen et al. for aortic velocity metrics.

In our study, FlowVN performed excellently at reconstructing not only data acquired with the same sequence at the same scanner as the training data but also data from a different site. This highlights the generalizability of FlowVN even in the presence of variations in acquisition protocol and its suitability for multi‐center studies and clinical applications. Quantitative results show that flow volume estimation in the AAo in Site A data tended to deviate from the ground truth, especially at the higher acceleration factors. However, since the model used to reconstruct the images were trained at data from Site A and the corresponding issue was not seen in the data from Site B, the reduced quality of flow rate estimation at very high acceleration factors may be a result of the quality of the tested Site A data due to breathing artifacts, rather than a performance issue related to FlowVN. Future work will include reducing respiratory motion artifacts in the fully sampled training reference data by acquiring the 4D Flow data in a coronal orientation, and by exploring non‐Cartesian sampling strategies, such as 3D radial trajectories, to improve robustness to motion.

A novel contribution of this study is the evaluation of TKE, offering insights into turbulent flow characteristics. In spite of being evaluated in normal volunteers, where TKE is overall low, the magnitude‐based TKE was well preserved up to acceleration factors of 10 but deviated more clearly from the ground truth and had larger inter‐model variation at high acceleration factors > 12, potentially reflecting the fact that higher acceleration factors lead to lower SNR and thereby a narrower TKE dynamic range [39]. It should be noted that TKE in the Site B data is low, and sometimes even artifactually negative, when compared to values reported in the literature for normal volunteers [40, 41], which might have exaggerated this effect. Notably, in volunteers with higher TKE, such as Volunteer 5 from Site A (Figure S4B) and Volunteer 1 from Site B (Figure S4F), the reconstructed TKE values agreed well with ground truth even at acceleration factors up to 12. Further, in aortic stenosis patients with higher TKE, FlowVN reconstructed TKE was well preserved even at acceleration factors up to 22. Low TKE is of limited clinical significance, and the relatively poor performance at low TKE is therefore of limited concern, especially in light of the performance at higher TKE. Nevertheless, further studies in prospectively undersampled data with elevated TKE are necessary to validate these results.

### 4.1 Limitations

A limitation of this study is that FlowVN reconstructions were not compared against conventional CS‐based reconstructions. However, FlowVN has previously been shown to perform equally well or better than CS [22]. Here, we focused on a detailed investigation of the performance of FlowVN on multi‐site data across a broad range of acceleration factors and relevant hemodynamic parameters. Additionally, while we demonstrated that a FlowVN model trained on a single dataset successfully reconstructs data from multiple sites, further evaluations are needed to explore the limits of FlowVN's generalizability. This entails investigations on data from multiple vendors, a wider variety of 4D Flow MRI sequences, and applications beyond the aorta. Finally, this study employed retrospectively undersampled data, a small set of publicly available prospectively undersampled data along with the use of CS reconstructed data as a starting point for subsequent undersampling and evaluation of FlowVN reconstructions in aortic stenosis patients. Parallel imaging reference data in AS patients were not available, which limited our ability to use low‐acceleration acquisitions as ground truth. Evaluations with prospectively undersampled data from multiple sites are needed to further assess the performance of FlowVN.

## 5 Conclusion

FlowVN trained on a single 4D Flow MRI dataset is capable of reconstructing highly undersampled 4D Flow MRI data from multiple sites at a quality that permits accurate quantification of peak velocity and flow volumes at high acceleration factors. In addition to excellent performance for velocity‐based parameters, FlowVN reconstructed 4D Flow MR shows potential to estimate TKE at high acceleration factors in patients with elevated TKE.

## Funding

This work was supported by the strategic research area in circulation and metabolism at Linköping University. CircM is a strategic research network for researchers at Linköping University and Region Östergötland within the research field circulation and cardio‐metabolic risk factors. https://liu.se/en/research/liu‐circm.

ALF Grants, Region Östergötland. https://www.regionostergotland.se/ro/det‐har‐gor‐vi/forskning/for‐dig‐som‐forskar/ansok‐om‐forskningsmedel.

Vetenskapsrådet.

Analytic Imaging Diagnostics Arena. AIDA is a national arena for research and innovation in medical image analysis. AIDA is a multidisciplinary collaboration aimed at large‐scale use of AI in healthcare. In this arena, academia, healthcare and industry come together to turn technical advances in AI technology into patient benefits in the form of clinically useful tools. https://liu.se/en/research/aida.

## Supporting information


**Figure S1:** 4D Flow magnitude images from Site A and B, along with visualization of flow in the segmented aorta. The planes indicate the positions for flow calculation in the ascending and descending aorta.
**Figure S2:** Site‐A volunteer results are summarized as grouped box plots for nRMSE, Angular Error, and Relative Error. Within each training type (Training Set 1—Training Set 5; models trained on 1–5 datasets, respectively), distributions are shown for each acceleration factor (AF 6, 10, 14, 18, 22). For each AF, in each training set, there are three boxes corresponding to the three independently trained models with that training‐set size.
**Figure S3:** Comparison of coefficient of variation across the 15 models for different performance metrics at different acceleration factors for all the test volunteers. FR = Flow rate, FV = Flow volume, AAo = Ascending aorta, DAo = descending aorta.
**Figure S4:** Plots of total TKE in the thoracic aorta for three representative cases (best, median, worst) from Site A and Site B datasets (Volunteers) at various acceleration factors, reconstructed with a model obtained by training with a single Site A dataset.
**Figure S5:** Example of ground truth and prospectively accelerated FlowVN‐reconstructed magnitude and foot‐to‐head (FH) velocity images for six volunteers from Site B. Acceleration factor ranges from 12.4 to 13.8. The FlowVN model was trained with a single dataset from Site A.
**Table S1:** Mean and Standard Deviation of average, maximum velocities and total TKE for data reconstructed using 15 models from six healthy volunteers from each site (A and B) at various acceleration factors. Data are reported as Mean ± SD.
**Table S2:** Mean and Standard Deviation for nRMSE, angular error and relative error for data reconstructed using 15 models from 6 healthy volunteers from each site (A and B) at various acceleration factors. Data are reported as Mean ± SD.
**Table S3:** Mean and Standard Deviation for flow rate (mL/s) in ascending aorta (AAo) and descending aorta (DAo) for data reconstructed using 15 models from 6 healthy volunteers from each site (A and B) at various acceleration factors. Data are reported as Mean ± SD.
**Table S4:** Mean and Standard Deviation for flow (mL) through planes in ascending aorta (AAo) and descending aorta (DAo) in one cardiac cycle for data reconstructed using 15 models from 6 healthy volunteers from each site (A and B) at various acceleration factors. Data are reported as Mean ± SD.
**Table S5:** nRMSE, angular error and relative error for data reconstructed from 6 healthy volunteers from each site (A and B) using a model trained with single dataset at various acceleration factors.
**Table S6:** Difference in average total TKE (mJ) between reconstructed data and ground truth for 6 healthy volunteers from each site (A and B) using a model trained with single dataset at various acceleration factors (AF).

## Data Availability

The data that support the findings of this study will be openly available in 4DFlowVN Cross Site Rapid CMR at https://gitlab.liu.se/cim_public/4dflowvn‐cross‐site‐rapid‐cmr after publication.
