# Supplementary material for: FlowVN Trained on a Single Dataset Enables Rapid Reconstruction of Highly Accelerated 4D Flow MRI Across Multiple Sites
Source: Magn Reson Med. 2026 Feb 26;96(1):374–86. doi: 10.1002/mrm.70317 (PMC13156433; doi:10.1002/mrm.70317)
Supplement: Supplementary file 1 — Figure S1: 4D Flow magnitude images from Site A and B, along with visualization of flow in the segmented aorta. The planes indicate the positions for flow calculation in the ascending and descending aorta. Figure S2: Site‐A volunteer results are summarized as grouped box plots for nRMSE, Angular Error, and Relative Error. Within each training type (Training Set 1—Training Set 5; models trained on 1–5 datasets, respectively), distributions are shown for each acceleration factor (AF 6, 10, 14, 18, 22). For each AF, in each training set, there are three boxes corresponding to the three independently trained models with that training‐set size. Figure S3: Comparison of coefficient of variation across the 15 models for different performance metrics at different acceleration factors for all the test volunteers. FR = Flow rate, FV = Flow volume, AAo = Ascending aorta, DAo = descending aorta. Figure S4: Plots of total TKE in the thoracic aorta for three representative cases (best, median, worst) from Site A and Site B datasets (Volunteers) at various acceleration factors, reconstructed with a model obtained by training with a single Site A dataset. Figure S5: Example of ground truth and prospectively accelerated FlowVN‐reconstructed magnitude and foot‐to‐head (FH) velocity images for six volunteers from Site B. Acceleration factor ranges from 12.4 to 13.8. The FlowVN model was trained with a single dataset from Site A. Table S1: Mean and Standard Deviation of average, maximum velocities and total TKE for data reconstructed using 15 models from six healthy volunteers from each site (A and B) at various acceleration factors. Data are reported as Mean ± SD. Table S2: Mean and Standard Deviation for nRMSE, angular error and relative error for data reconstructed using 15 models from 6 healthy volunteers from each site (A and B) at various acceleration factors. Data are reported as Mean ± SD. Table S3: Mean and Standard Deviation for flow rate (mL/s) in ascending aorta (AAo) and d [file MRM-96-374-s001.docx]

**Appendix**


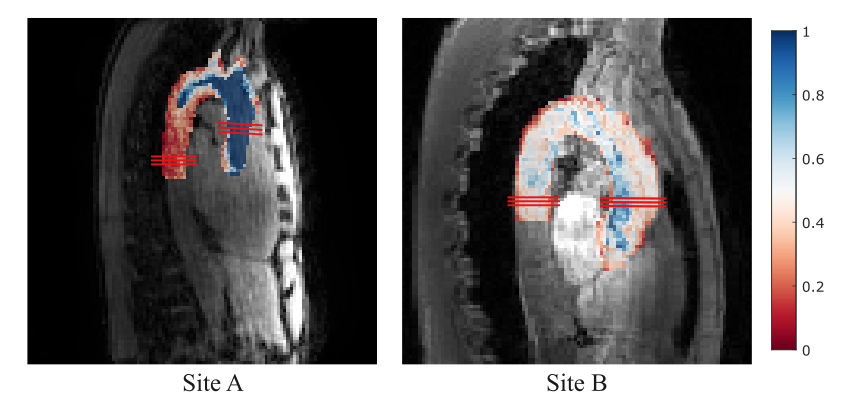


Figure S1: 4D Flow magnitude images from Site A and B, along with visualization of flow in the segmented aorta. The planes indicate the positions for flow calculation in the ascending and descending aorta.


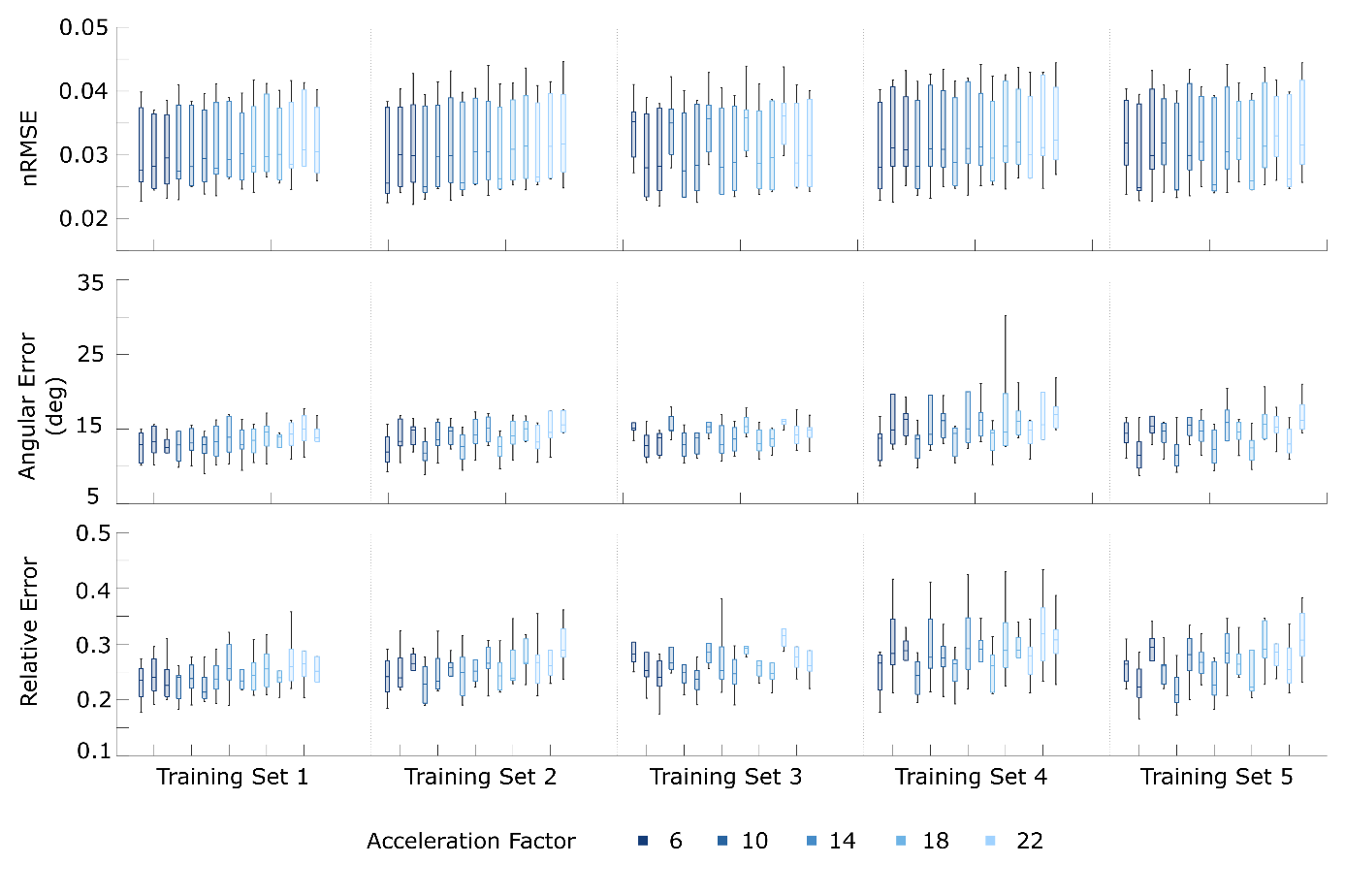


Figure S2: Site-A volunteer results are summarized as grouped box plots for nRMSE, Angular Error, and Relative Error. Within each training type (Training Set 1 – Training Set 5; models trained on 1–5 datasets, respectively), distributions are shown for each acceleration factor (AF 6, 10, 14, 18, 22). For each AF, in each training set, there are three boxes corresponding to the three independently trained models with that training-set size.


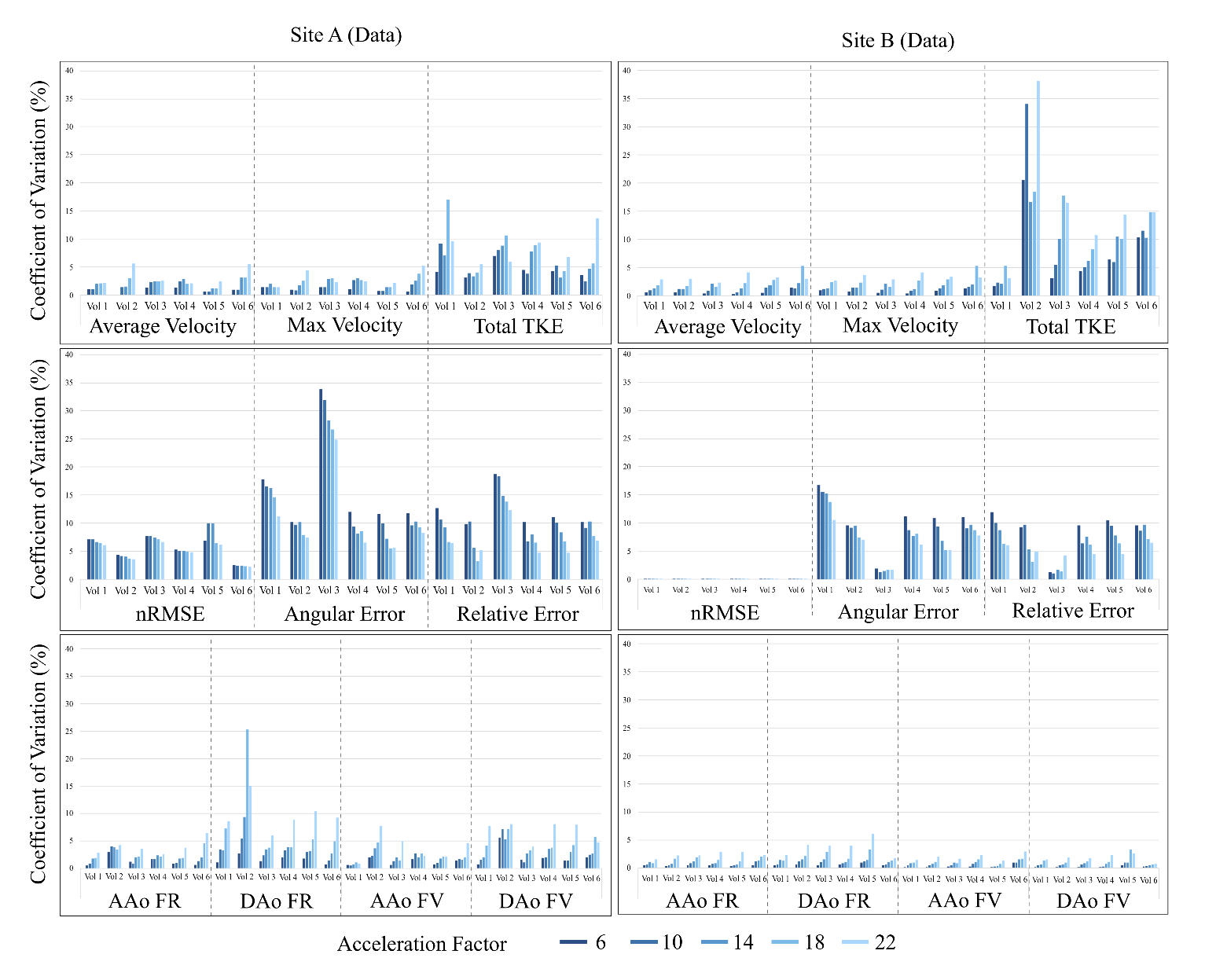


Figure S3: Comparison of coefficient of variation across the 15 models for different performance metrics at different acceleration factors for all the test volunteers. FR = Flow rate, FV = Flow volume, AAo = Ascending aorta, DAo = descending aorta.


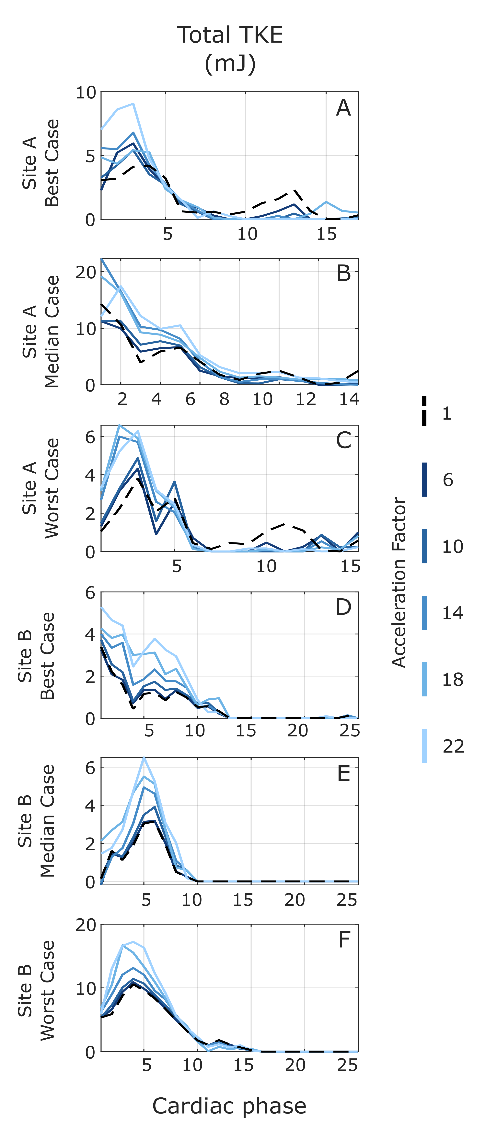


Figure S4: Plots of total TKE in the thoracic aorta for three representative cases (best, median, worst) from Site A and Site B datasets (Volunteers) at various acceleration factors, reconstructed with a model obtained by training with a single Site A dataset.


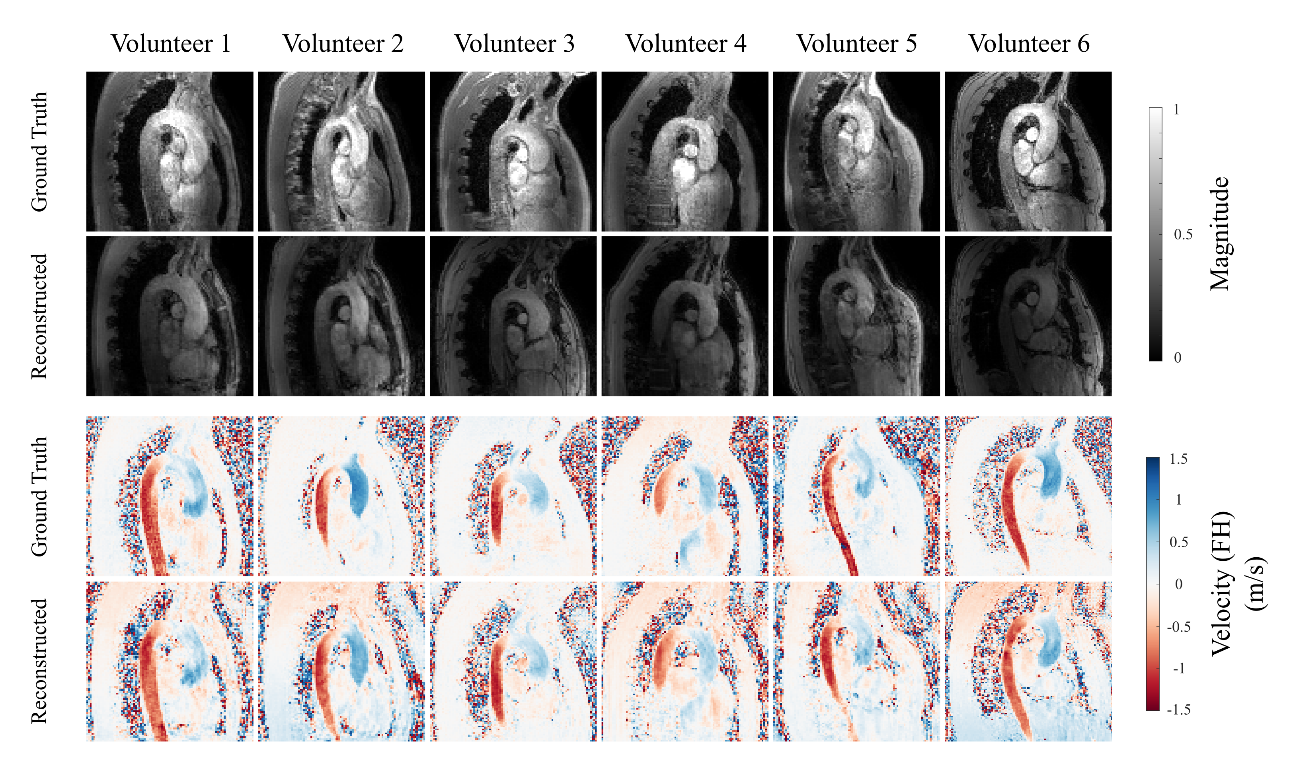


Figure S5: Example of ground truth and prospectively accelerated FlowVN-reconstructed magnitude and foot-to-head (FH) velocity images for six volunteers from Site B. Acceleration factor ranges from 12.4 to 13.8. The FlowVN model was trained with a single dataset from Site A.

Table S1: Mean and Standard Deviation of average, maximum velocities and total TKE for data reconstructed using 15 models from six healthy volunteers from each site (A and B) at various acceleration factors. Data are reported as Mean ± SD.

|  | | **Site A Data** | | | | | | **Site B Data** | | | | | |
| --- | --- | --- | --- | --- | --- | --- | --- | --- | --- | --- | --- | --- | --- |
| **Acceleration**  **Factor** | | **1** | **6** | **10** | **14** | **18** | **22** | **1** | **6** | **10** | **14** | **18** | **22** |
| **Average Velocity [m/s]** | Vol 1 | 0.72 ± 0 | 0.72 ± .003 | 0.73 ± 0.005 | 0.73 ± .008 | 0.74 ± 0.012 | 0.67 ± 0.018 | 0.78 ± 0 | 0.78 ± 0.003 | 0.77 ± 0.004 | 0.76 ± 0.006 | 0.75 ± 0.012 | 0.74 ± 0.017 |
|  | Vol 2 | 0.71 ± 0 | 0.71 ± .004 | 0.68 ± 0.006 | 0.61 ± .014 | 0.57 ± 0.012 | 0.52 ± 0.028 | 0.85 ± 0 | 0.84 ± 0.002 | 0.84 ± 0.003 | 0.84 ± 0.004 | 0.83 ± 0.008 | 0.83 ± 0.014 |
|  | Vol 3 | 0.70 ± 0 | 0.69 ± .002 | 0.69 ± 0.004 | 0.65 ± .006 | 0.64 ± 0.006 | 0.66 ± 0.013 | 0.71 ± 0 | 0.71 ± 0.002 | 0.71 ± 0.003 | 0.70 ± 0.006 | 0.69 ± 0.009 | 0.68 ± 0.013 |
|  | Vol 4 | 0.78 ± 0 | 0.79 ± .002 | 0.77 ± 0.005 | 0.73 ± .007 | 0.70 ± 0.011 | 0.72 ± 0.014 | 0.70 ± 0 | 0.69 ± 0.001 | 0.70 ± 0.004 | 0.69 ± 0.004 | 0.69 ± 0.007 | 0.66 ± 0.012 |
|  | Vol 5 | 0.78 ± 0 | 0.79 ± .002 | 0.77 ± 0.005 | 0.73 ± .007 | 0.70 ± 0.011 | 0.72 ± 0.014 | 0.60 ± 0 | 0.59 ± 0.002 | 0.59 ± 0.002 | 0.59 ± 0.004 | 0.58 ± 0.006 | 0.57 ± 0.01 |
|  | Vol 6 | 0.65 ± 0 | 0.66 ± .003 | 0.65 ± 0.004 | 0.60 ± .006 | 0.57 ± 0.009 | 0.53 ± 0.014 | 0.26 ± 0 | 0.26 ± 0.002 | 0.25 ± 0.004 | 0.25 ± 0.004 | 0.26 ± 0.007 | 0.27 ± 0.008 |
| **Max Velocity [m/s]** | Vol 1 | 1.08 ±0 | 1.08 ± .006 | 1.09 ± 0.009 | 1.06 ± 0.011 | 1.10 ± 0.019 | 1.05 ± 0.022 | 1.27 ± 0 | 1.26 ± 0.006 | 1.26 ± 0.006 | 1.23 ± 0.005 | 1.20 ± 0.018 | 1.18 ± 0.023 |
|  | Vol 2 | 1.09 ± 0 | 1.09 ± .006 | 1.08 ± 0.011 | 0.97 ± .019 | 0.92 ± 0.016 | 0.87 ± 0.057 | 1.13 ± 0 | 1.13 ± 0.003 | 1.12 ± 0.005 | 1.10 ± 0.003 | 1.10 ± 0.008 | 1.09 ± 0.017 |
|  | Vol 3 | 1.07 ± 0 | 1.02 ± .006 | 1.01 ± 0.009 | 0.95 ± .008 | 0.94 ± 0.009 | 1.00 ± 0.02 | 1.12 ± 0 | 1.12 ± 0.003 | 1.11 ± 0.006 | 1.10 ± 0.01 | 1.08 ± 0.016 | 1.07 ± 0.022 |
|  | Vol 4 | 1.20 ± 0 | 1.20 ± .004 | 1.16 ± 0.006 | 1.10 ± 0.011 | 1.07 ± 0.019 | 1.18 ± 0.024 | 1.03 ± 0 | 1.02 ± 0.003 | 1.02 ± 0.007 | 1.02 ± 0.007 | 1.01 ± 0.011 | 0.97 ± 0.025 |
|  | Vol 5 | 1.20 ± 0 | 1.20 ± .004 | 1.16 ± 0.006 | 1.10 ± 0.011 | 1.07 ± 0.019 | 1.18 ± 0.024 | 0.93 ± 0 | 0.92 ± 0.003 | 0.91 ± 0.004 | 0.92 ± 0.008 | 0.90 ± 0.012 | 0.91 ± 0.017 |
|  | Vol 6 | 0.99 ± 0 | 0.97 ± .005 | 0.94 ± 0.007 | 0.90 ± .008 | 0.84 ± 0.015 | 0.84 ± 0.017 | 0.47 ± 0 | 0.47 ± 0.004 | 0.44 ± 0.008 | 0.45 ± 0.007 | 0.46 ± 0.012 | 0.47 ± 0.015 |
| **Total TKE**  **[mJ]** | Vol 1 | 4.10 ± 0 | 5.40 ± .200 | 4.40 ± 0.400 | 5.90 ± 0.40 | 3.70 ± 0.600 | 9.00 ± 0.900 | 10.6 ± 0 | 11.00 ± 0.20 | 11.5 ± 0.300 | 13.50 ± 0.30 | 14.50 ± 0.80 | 16.70 ± 0.50 |
|  | Vol 2 | 3.80 ± 0 | 4.10 ± .100 | 4.60 ± 0.200 | 5.60 ± 0.20 | 5.60 ± 0.200 | 5.70 ± 0.300 | 0.40 ± 0 | .60 ± 0.100 | 0.70 ± 0.200 | 1.30 ± 0.200 | 2.00 ± 0.400 | 1.40 ± 0.500 |
|  | Vol 3 | 1.50 ± 0 | 2.00 ± .100 | 1.90 ± 0.200 | 2.70 ± 0.20 | 2.40 ± 0.300 | 4.30 ± 0.300 | 3.00 ± 0 | 3.00 ± 0.100 | 3.30 ± 0.200 | 4.10 ± 0.400 | 4.20 ± 0.800 | 4.80 ± 0.800 |
|  | Vol 4 | 2.20 ± 0 | 5.40 ± .200 | 5.30 ± 0.200 | 5.20 ± 0.40 | 3.70 ± 0.300 | 5.80 ± 0.500 | 3.80 ± 0 | 4.20 ± 0.200 | 4.70 ± 0.200 | 5.60 ± 0.300 | 5.80 ± 0.500 | 6.20 ± 0.700 |
|  | Vol 5 | 4.00 ± 0 | 6.20 ± .300 | 7.30 ± 0.400 | 10.4 ± 0.30 | 9.10 ± 0.400 | 13.10 ± 0.90 | 1.50 ± 0 | 1.60 ± 0.100 | 2.00 ± 0.100 | 2.20 ± 0.200 | 2.70 ± 0.300 | 3.10 ± 0.400 |
|  | Vol 6 | 3.00 ± 0 | 3.90 ± .100 | 4.50 ± 0.100 | 4.10 ± 0.20 | 4.90 ± 0.300 | 5.60 ± 0.800 | 1.70 ± 0 | 1.90 ± 0.200 | 2.10 ± 0.200 | 3.00 ± 0.300 | 3.60 ± 0.500 | 4.00 ± 0.600 |

Table S2: Mean and Standard Deviation for nRMSE, angular error and relative error for data reconstructed using 15 models from 6 healthy volunteers from each site (A and B) at various acceleration factors. Data are reported as Mean ± SD.

|  | | **Site A Data** | | | | | **Site B Data** | | | | |
| --- | --- | --- | --- | --- | --- | --- | --- | --- | --- | --- | --- |
| **Acceleration  Factor** | | **6** | **10** | **14** | **18** | **22** | **6** | **10** | **14** | **18** | **22** |
| **nRMSE** | Vol 1 | 0.03 ± 0.002 | 0.03 ± 0.002 | 0.03 ± 0.002 | 0.03 ± 0.002 | 0.03 ± 0.002 | 0.22 ± 0 | 0.22 ± 0 | 0.22 ± 0 | 0.22 ± 0 | 0.22 ± 0 |
|  | Vol 2 | 0.02 ± 0.001 | 0.02 ± 0.001 | 0.03 ± 0.001 | 0.03 ± 0.001 | 0.03 ± 0.001 | 0.21 ± 0 | 0.21 ± 0 | 0.21 ± 0 | 0.21 ± 0 | 0.21 ± 0 |
|  | Vol 3 | 0.03 ± 0.002 | 0.03 ± 0.002 | 0.03 ± 0.002 | 0.03 ± 0.002 | 0.03 ± 0.002 | 0.22 ± 0 | 0.22 ± 0 | 0.22 ± 0 | 0.22 ± 0 | 0.22 ± 0 |
|  | Vol 4 | 0.04 ± 0.002 | 0.04 ± 0.002 | 0.04 ± 0.002 | 0.04 ± 0.002 | 0.04 ± 0.002 | 0.22 ± 0 | 0.22 ± 0 | 0.22 ± 0 | 0.22 ± 0 | 0.22 ± 0 |
|  | Vol 5 | 0.03 ± 0.002 | 0.03 ± 0.003 | 0.03 ± 0.003 | 0.03 ± 0.002 | 0.03 ± 0.002 | 0.21 ± 0 | 0.21 ± 0 | 0.21 ± 0 | 0.21 ± 0 | 0.21 ± 0 |
|  | Vol 6 | 0.04 ± 0.001 | 0.04 ± 0.001 | 0.04 ± 0.001 | 0.04 ± 0.001 | 0.04 ± 0.001 | 0.21 ± 0 | 0.21 ± 0 | 0.21 ± 0 | 0.21 ± 0 | 0.21 ± 0 |
| **Ang Err** | Vol 1 | 11.3 ± 2.00 | 11.6 ± 1.9 | 12.4 ± 2.00 | 12.8 ± 1.9 | 14.3 ± 1.6 | 7.7 ± 0.1 | 10.3 ± 0.1 | 13.8 ± 0.1 | 16.1 ± 0.3 | 18.0 ± 0.3 |
|  | Vol 2 | 15.6 ± 1.6 | 16.7 ± 1.6 | 17.3 ± 1.8 | 19.6 ± 1.6 | 21.6 ± 1.6 | 4.2 ± 0.1 | 5.6 ± 0.1 | 6.6 ± 0.1 | 7.5 ± 0.1 | 8.2 ± 0.1 |
|  | Vol 3 | 15.1 ± 5.1 | 15.3 ± 4.9 | 16.3 ± 4.6 | 16.8 ± 4.5 | 17.5 ± 4.4 | 5.4 ± 0.1 | 6.9 ± 0.1 | 8.3 ± 0.1 | 9.5 ± 0.2 | 10.7 ± 0.2 |
|  | Vol 4 | 14.7 ± 1.8 | 14.2 ± 1.3 | 15.3 ± 1.2 | 15.9 ± 1.4 | 17.3 ± 1.1 | 6.3 ± 0.1 | 8.5 ± 0.1 | 10.8 ± 0.2 | 12.7 ± 0.2 | 14.4 ± 0.3 |
|  | Vol 5 | 12.2 ± 1.4 | 12.6 ± 1.3 | 14.1 ± 1.00 | 15.3 ± 0.8 | 16.3 ± 0.90 | 8.1 ± 0.1 | 10.5 ± 0.1 | 12.3 ± 0.1 | 14.0 ± 0.2 | 15.0 ± 0.2 |
|  | Vol 6 | 14.7 ± 1.7 | 15.9 ± 1.5 | 15.7 ± 1.6 | 16.5 ± 1.5 | 18.4 ± 1.5 | 10.8 ± 0.1 | 12.1 ± 0.1 | 15.0 ± 0.2 | 17.1 ± 0.2 | 18.5 ± 0.3 |
| **Rel Err** | Vol 1 | 0.24 ± 0.03 | 0.24 ± 0.026 | 0.26 ± 0.024 | 0.29 ± 0.019 | 0.33 ± 0.021 | 0.08 ± 0.001 | 0.11 ± 0.001 | 0.15 ± 0.003 | 0.18 ± 0.006 | 0.22 ± 0.006 |
|  | Vol 2 | 0.31 ± 0.03 | 0.32 ± 0.033 | 0.37 ± 0.021 | 0.43 ± 0.014 | 0.44 ± 0.023 | 0.06 ± 0 | 0.08 ± 0.001 | 0.1 ± 0.002 | 0.13 ± 0.002 | 0.14 ± 0.006 |
|  | Vol 3 | 0.27 ± 0.051 | 0.28 ± 0.051 | 0.3 ± 0.045 | 0.31 ± 0.043 | 0.33 ± 0.041 | 0.07 ± 0.001 | 0.09 ± 0.001 | 0.11 ± 0.002 | 0.13 ± 0.002 | 0.15 ± 0.007 |
|  | Vol 4 | 0.25 ± 0.026 | 0.25 ± 0.017 | 0.27 ± 0.022 | 0.27 ± 0.018 | 0.3 ± 0.014 | 0.07 ± 0.001 | 0.09 ± 0.001 | 0.12 ± 0.003 | 0.16 ± 0.006 | 0.2 ± 0.015 |
|  | Vol 5 | 0.2 ± 0.022 | 0.21 ± 0.021 | 0.23 ± 0.019 | 0.25 ± 0.017 | 0.3 ± 0.014 | 0.12 ± 0.002 | 0.15 ± 0.002 | 0.18 ± 0.003 | 0.21 ± 0.005 | 0.23 ± 0.011 |
|  | Vol 6 | 0.28 ± .028 | 0.28 ± 0.026 | 0.29 ± 0.03 | 0.33 ± 0.025 | 0.38 ± 0.026 | 0.13 ± 0.001 | 0.15 ± 0.002 | 0.18 ± 0.004 | 0.21 ± 0.008 | 0.23 ± 0.006 |

Table S3: Mean and Standard Deviation for flow rate (mL/s) in ascending aorta (AAo) and descending aorta (DAo) for data reconstructed using 15 models from 6 healthy volunteers from each site (A and B) at various acceleration factors. Data are reported as Mean ± SD.

|  | | **Site A Data** | | | | | | **Site B Data** | | | | | |
| --- | --- | --- | --- | --- | --- | --- | --- | --- | --- | --- | --- | --- | --- |
| **Acceleration**  **Factor** | | **1** | **6** | **10** | **14** | **18** | **22** | **1** | **6** | **10** | **14** | **18** | **22** |
| **AAo Flow Rate [mL/s]** | Vol 1 | 232.2 ± 0 | 230.9 ± 1.0 | 238.7 ± 2.0 | 212.6 ± 3.7 | 207.8 ± 3.9 | 200.2 ± 5.5 | 526.5 ± 0 | 560.7 ± 2.4 | 567.3 ± 3.6 | 556.8 ± 5.6 | 565. ± 4.4 | 554.6 ± 8.2 |
|  | Vol 2 | 163.6 ± 0 | 173.2 ± 5.0 | 203.1 ± 8.0 | 176.9 ± 6.7 | 198.4 ± 6.8 | 170.7 ± 7.2 | 304.2 ± 0 | 315.8 ± 1.3 | 315.9 ± 1.6 | 311.4 ± 2.3 | 311.1 ± 5.2 | 302.9 ± 6.6 |
|  | Vol 3 | 266.1 ± 0 | 280.2 ± 3.2 | 252.1 ± 2.0 | 261.1 ± 5.2 | 250. ± 5.1 | 248.5 ± 8.7 | 213.8 ± 0 | 214.4 ± 1.1 | 213.3 ± 1.8 | 205.2 ± 2.3 | 206.4 ± 3.7 | 211. ± 4.8 |
|  | Vol 4 | 192.6 ± 0 | 206.8 ± 3.4 | 219. ± 3.6 | 208.1 ± 5.0 | 193.6 ± 4.0 | 183.7 ± 4.7 | 231.5 ± 0 | 235.4 ± 1.2 | 237.7 ± 1.5 | 233.1 ± 2.0 | 233.6 ± 3.2 | 225.2 ± 6.3 |
|  | Vol 5 | 252.6 ± 0 | 255.5 ± 2.0 | 244.7 ± 2.4 | 242. ± 4.3 | 221.8 ± 4.2 | 208.3 ± 7.8 | 219.4 ± 0 | 212.2 ± 0.7 | 215.1 ± 1.1 | 212.8 ± 1.3 | 216.1 ± 2.6 | 216.2 ± 6.1 |
|  | Vol 6 | 191.7 ± 0 | 193.8 ± 1.2 | 188.4 ± 2.5 | 183.6 ± 3.7 | 166.6 ± 7.6 | 162.9 ± 10.4 | 189.1 ± 0 | 211.9 ± 1.0 | 211.4 ± 2.4 | 212.6 ± 2.8 | 202.8 ± 4.0 | 210.9 ± 4.9 |
| **DAo Flow Rate**  **[mL/s]** | Vol 1 | 283.4 ± 0 | 243.6 ± 2.4 | 276.6 ± 9.3 | 278.7 ± 9.3 | 282.8 ± 20.4 | 226.2 ± 19.2 | 363.6 ± 0 | 328.6 ± 1.6 | 328.4 ± 1.9 | 323.7 ± 4.5 | 307.2 ± 3.9 | 314.3 ± 7.3 |
|  | Vol 2 | 172.3 ± 0 | 204.6 ± 5.4 | 143.2 ± 7.6 | 138.3 ± 12.9 | 37. ± 9.4 | 115.2 ± 17.9 | 202.1 ± 0 | 187.4 ± 1.2 | 185.5 ± 2.1 | 190.3 ± 2.8 | 178.6 ± 3.7 | 179.5 ± 7.3 |
|  | Vol 3 | 176.4 ± 0 | 171.7 ± 2.1 | 181.9 ± 4.2 | 152.1 ± 5.2 | 152.2 ± 5.7 | 163.2 ± 9.6 | 181.4 ± 0 | 160.8 ± 0.7 | 159.9 ± 1.6 | 164.3 ± 2.6 | 153.7 ± 4.2 | 156. ± 6.2 |
|  | Vol 4 | 113.6 ± 0 | 108.6 ± 2.1 | 103.9 ± 3.4 | 113.5 ± 4.4 | 115.7 ± 4.5 | 115.1 ± 10.1 | 285.5 ± 0 | 257.4 ± 1.5 | 257.3 ± 2.2 | 253.9 ± 2.8 | 256.8 ± 4.2 | 236.3 ± 9.3 |
|  | Vol 5 | 196.4 ± 0 | 217.1 ± 3.8 | 204.1 ± 5.9 | 169. ± 5.2 | 163.6 ± 8.5 | 176. ± 18.3 | 147.6 ± 0 | 130.7 ± 1.2 | 131.4 ± 1.6 | 121.6 ± 1.8 | 124.5 ± 4.1 | 111.7 ± 6.7 |
|  | Vol 6 | 133.7 ± 0 | 132.6 ± 0.9 | 131.4 ± 1.8 | 110.6 ± 2.9 | 115.7 ± 5.7 | 79. ± 7.2 | 239.5 ± 0 | 262.8 ± 1.2 | 259.2 ± 1.4 | 257.7 ± 2.6 | 254.1 ± 3.2 | 257.6 ± 4.7 |

Table S4: Mean and Standard Deviation for flow (mL) through planes in ascending aorta (AAo) and descending aorta (DAo) in one cardiac cycle for data reconstructed using 15 models from 6 healthy volunteers from each site (A and B) at various acceleration factors. Data are reported as Mean ± SD.

|  | | **Site A Data** | | | | | | **Site B Data** | | | | | |
| --- | --- | --- | --- | --- | --- | --- | --- | --- | --- | --- | --- | --- | --- |
| **Acceleration**  **Factor** | | **1** | **6** | **10** | **14** | **18** | **22** | **1** | **6** | **10** | **14** | **18** | **22** |
| **AAo Flow [mL]** | Vol 1 | 83.3 ± 0 | 80.1 ± .50 | 80.3 ± 0.40 | 79.2 ± 0.60 | 78.7 ± 0.80 | 77.6 ± 0.60 | 93.8 ± 0 | 104.6 ± 0.20 | 105.6 ± 0.40 | 102.5 ± 0.90 | 103.1 ± 0.90 | 105.3 ± 1.40 |
|  | Vol 2 | 50.3 ± 0 | 55.3 ± 1.1 | 52.5 ± 1.2 | 48.5 ± 1.8 | 41.1 ± 1.9 | 34.5 ± 2.7 | 71.7 ± 0 | 70. ± 0.10 | 70.6 ± 0.30 | 69.5 ± 0.50 | 69.4 ± 0.70 | 70.6 ± 1.40 |
|  | Vol 3 | 59.4 ± 0 | 58.2 ± 0.40 | 58. ± 0.70 | 59.6 ± 1.2 | 59.4 ± 0.80 | 56.2 ± 2.8 | 61.3 ± 0 | 64.3 ± 0.20 | 64.3 ± 0.30 | 63.1 ± 0.60 | 63.9 ± 0.60 | 63.1 ± 1.10 |
|  | Vol 4 | 54.3 ± 0 | 54.4 ± 0.90 | 54.1 ± 1.4 | 54.3 ± 1.1 | 46.8 ± 1.3 | 48.2 ± 1.1 | 61.4 ± 0 | 63.2 ± 0.20 | 65. ± 0.50 | 62.3 ± 0.60 | 62.6 ± 0.90 | 57.4 ± 1.30 |
|  | Vol 5 | 71.9 ± 0 | 69.5 ± 0.50 | 69.3 ± 0.70 | 68.9 ± 1.2 | 60.8 ± 1.3 | 57.7 ± 1.2 | 67.3 ± 0 | 63.5 ± 0.10 | 63.8 ± 0.20 | 63.8 ± 0.30 | 64.1 ± 0.40 | 63.4 ± 0.80 |
|  | Vol 6 | 42. ± 0 | 39.9 ± 0.60 | 40.2 ± 0.70 | 40. ± 0.60 | 37.3 ± 0.70 | 37.6 ± 1.7 | 95. ± 0 | 93.4 ± 0.80 | 95.2 ± 0.90 | 89.9 ± 1.40 | 88.5 ± 1.50 | 93.7 ± 2.70 |
| **DAo Flow [mL]** | Vol 1 | 60.6 ± 0 | 60.4 ± 0.50 | 65.8 ± 1.0 | 67.6 ± 1.4 | 69.7 ± 2.8 | 63.9 ± 4.9 | 59.9 ± 0 | 58. ± 0.10 | 58.1 ± 0.20 | 57.5 ± 0.30 | 54.7 ± 0.70 | 57.0 ± 0.90 |
|  | Vol 2 | 42.3 ± 0 | 44.5 ± 2.5 | 37.3 ± 2.7 | 35.8 ± 1.9 | 23.2 ± 1.7 | 35.5 ± 2.9 | 41.7 ± 0 | 40. ± 0.00 | 39.9 ± 0.20 | 40.7 ± 0.30 | 39.4 ± 0.40 | 38.5 ± 0.70 |
|  | Vol 3 | 30.5 ± 0 | 33.7 ± 0.50 | 34.6 ± 0.40 | 30.5 ± 0.80 | 31.6 ± 1.0 | 33. ± 1.3 | 49.6 ± 0 | 43.8 ± 0.10 | 44.4 ± 0.30 | 44.6 ± 0.40 | 42.4 ± 0.50 | 43.3 ± 0.80 |
|  | Vol 4 | 28.8 ± 0 | 26.8 ± 0.50 | 26.7 ± 0.50 | 24.5 ± 0.90 | 31.4 ± 1.2 | 25.9 ± 2.1 | 52.9 ± 0 | 51.8 ± 0.10 | 52. ± 0.20 | 50.7 ± 0.30 | 51.7 ± 0.50 | 50.0 ± 1.10 |
|  | Vol 5 | 63.1 ± 0 | 63.7 ± 0.90 | 63.4 ± 0.90 | 57.8 ± 1.7 | 46.8 ± 1.9 | 44.8 ± 3.5 | 42.9 ± 0 | 36.7 ± 0.20 | 37. ± 0.40 | 37.6 ± 0.30 | 36.4 ± 1.20 | 36.7 ± 1.00 |
|  | Vol 6 | 26.5 ± 0 | 28.1 ± 0.50 | 31. ± 0.80 | 31.2 ± 0.80 | 28.9 ± 1.7 | 27.9 ± 1.3 | 65.7 ± 0 | 72.7 ± 0.10 | 72.4 ± 0.20 | 71.3 ± 0.40 | 71.9 ± 0.50 | 71.6 ± 0.50 |

Table S5: nRMSE, angular error and relative error for data reconstructed from 6 healthy volunteers from each site (A and B) using a model trained with single dataset at various acceleration factors.

|  | **AF** | **Site A** | | | **Site B** | | |
| --- | --- | --- | --- | --- | --- | --- | --- |
|  |  | **nRMSE** | **Angular Error** | **Relative Error** | **nRMSE** | **Angular Error** | **Relative Error** |
| Vol 1 | 6 | 0.029 | 10.18 | 0.22 | 0.21747 | 7.82 | 0.08 |
|  | 8 | 0.029 | 10.03 | 0.22 | 0.21747 | 8.94 | 0.10 |
|  | 10 | 0.031 | 10.34 | 0.24 | 0.21747 | 10.46 | 0.11 |
|  | 12 | 0.031 | 10.32 | 0.22 | 0.21748 | 11.56 | 0.13 |
|  | 14 | 0.033 | 11.26 | 0.25 | 0.21748 | 13.67 | 0.15 |
|  | 16 | 0.033 | 11.21 | 0.27 | 0.21749 | 14.86 | 0.16 |
|  | 18 | 0.035 | 11.99 | 0.27 | 0.21749 | 16.59 | 0.19 |
|  | 20 | 0.036 | 12.03 | 0.27 | 0.2175 | 16.9 | 0.19 |
|  | 22 | 0.037 | 13.89 | 0.32 | 0.21749 | 18.39 | 0.23 |
| Vol 2 | 6 | 0.025 | 15.60 | 0.30 | 0.20935 | 4.16 | 0.06 |
|  | 8 | 0.025 | 15.07 | 0.28 | 0.20935 | 4.91 | 0.07 |
|  | 10 | 0.026 | 16.72 | 0.32 | 0.20935 | 5.60 | 0.08 |
|  | 12 | 0.027 | 15.41 | 0.32 | 0.20935 | 6.30 | 0.09 |
|  | 14 | 0.028 | 17.70 | 0.37 | 0.20936 | 6.54 | 0.10 |
|  | 16 | 0.031 | 17.50 | 0.36 | 0.20936 | 6.54 | 0.10 |
|  | 18 | 0.03 | 19.80 | 0.43 | 0.20936 | 7.65 | 0.13 |
|  | 20 | 0.032 | 18.77 | 0.38 | 0.20937 | 7.73 | 0.13 |
|  | 22 | 0.032 | 21.65 | 0.43 | 0.20936 | 8.24 | 0.15 |
| Vol 3 | 6 | 0.025 | 13.22 | 0.26 | 0.21998 | 5.13 | 0.07 |
|  | 8 | 0.025 | 13.09 | 0.24 | 0.21999 | 5.84 | 0.08 |
|  | 10 | 0.027 | 13.74 | 0.26 | 0.21999 | 6.68 | 0.09 |
|  | 12 | 0.027 | 13.84 | 0.26 | 0.21999 | 7.30 | 0.10 |
|  | 14 | 0.029 | 14.52 | 0.28 | 0.22000 | 7.98 | 0.11 |
|  | 16 | 0.03 | 15.38 | 0.29 | 0.22000 | 8.87 | 0.12 |
|  | 18 | 0.031 | 15.13 | 0.29 | 0.22000 | 9.20 | 0.14 |
|  | 20 | 0.033 | 15.88 | 0.32 | 0.22000 | 9.58 | 0.14 |
|  | 22 | 0.033 | 15.62 | 0.31 | 0.22001 | 10.72 | 0.16 |
| Vol 4 | 6 | 0.036 | 13.31 | 0.22 | 0.21947 | 6.38 | 0.07 |
|  | 8 | 0.038 | 13.15 | 0.23 | 0.21947 | 7.42 | 0.08 |
|  | 10 | 0.038 | 14.06 | 0.25 | 0.21947 | 8.58 | 0.09 |
|  | 12 | 0.040 | 15.40 | 0.25 | 0.21948 | 10.04 | 0.11 |
|  | 14 | 0.040 | 15.50 | 0.24 | 0.21948 | 11.18 | 0.12 |
|  | 16 | 0.041 | 16.31 | 0.26 | 0.21948 | 11.45 | 0.14 |
|  | 18 | 0.042 | 15.76 | 0.28 | 0.21949 | 12.99 | 0.17 |
|  | 20 | 0.044 | 15.65 | 0.27 | 0.21949 | 13.45 | 0.16 |
|  | 22 | 0.045 | 16.85 | 0.30 | 0.21949 | 14.50 | 0.21 |
| Vol 5 | 6 | 0.027 | 11.81 | 0.19 | 0.21152 | 7.87 | 0.13 |
|  | 8 | 0.027 | 12.17 | 0.19 | 0.21152 | 9.03 | 0.14 |
|  | 10 | 0.028 | 11.92 | 0.19 | 0.21152 | 10.34 | 0.16 |
|  | 12 | 0.028 | 12.71 | 0.21 | 0.21152 | 11.14 | 0.16 |
|  | 14 | 0.028 | 13.44 | 0.20 | 0.21153 | 12.09 | 0.18 |
|  | 16 | 0.029 | 14.33 | 0.24 | 0.21153 | 14.01 | 0.22 |
|  | 18 | 0.030 | 14.84 | 0.25 | 0.21153 | 14.14 | 0.22 |
|  | 20 | 0.030 | 15.14 | 0.24 | 0.21153 | 15 | 0.24 |
|  | 22 | 0.030 | 15.77 | 0.28 | 0.21153 | 15.62 | 0.24 |
| Vol 6 | 6 | 0.037 | 15.39 | 0.27 | 0.21322 | 10.79 | 0.13 |
|  | 8 | 0.038 | 15.49 | 0.26 | 0.21322 | 10.79 | 0.13 |
|  | 10 | 0.039 | 16.97 | 0.30 | 0.21322 | 12.09 | 0.16 |
|  | 12 | 0.041 | 17.14 | 0.28 | 0.21322 | 12.09 | 0.16 |
|  | 14 | 0.041 | 16.77 | 0.29 | 0.21323 | 14.98 | 0.19 |
|  | 16 | 0.044 | 17.25 | 0.32 | 0.21323 | 15.99 | 0.19 |
|  | 18 | 0.044 | 18.44 | 0.35 | 0.21324 | 17.59 | 0.23 |
|  | 20 | 0.045 | 17.17 | 0.35 | 0.21324 | 18.21 | 0.23 |
|  | 22 | 0.047 | 20.97 | 0.37 | 0.21324 | 19.25 | 0.24 |

Table S6: Difference in average total TKE (mJ) between reconstructed data and ground truth for 6 healthy volunteers from each site (A and B) using a model trained with single dataset at various acceleration factors (AF).

| **AF** | | **6** | **8** | **10** | **12** | **14** | **16** | **18** | **20** | **22** |
| --- | --- | --- | --- | --- | --- | --- | --- | --- | --- | --- |
| **Site A** | Vol 1 | 0.2868 | 0.3234 | 0.3428 | 0.2754 | -0.0039 | -0.3416 | -0.0562 | -0.3841 | -0.2435 |
|  | Vol 2 | 0.2009 | 0.4374 | 0.2547 | 0.1006 | -0.0454 | -0.0912 | -0.3192 | -0.0184 | -0.2286 |
|  | Vol 3 | 0.0809 | -0.0338 | 0.0119 | -0.1830 | -0.3901 | -0.3171 | -0.3102 | -0.4315 | -0.8810 |
|  | Vol 4 | -0.1119 | -0.3523 | -0.5609 | -0.7150 | -0.9690 | -1.1499 | -0.5888 | -1.2302 | -1.1594 |
|  | Vol 5 | 0.4739 | 0.3249 | 0.3410 | 0.0815 | -1.4414 | -1.2776 | -1.4791 | -1.4289 | -1.7869 |
|  | Vol 6 | -0.7376 | -0.8418 | -0.6420 | -0.6819 | -0.760 | -0.7064 | -1.4799 | -0.6893 | -1.0938 |
| **Site B** | Vol 1 | 0.0051 | 0.0055 | 0.0084 | 0.0025 | 0.0085 | 0.0031 | 0.0093 | 0.0117 | 0.0195 |
|  | Vol 2 | -0.0052 | -0.0654 | -0.1304 | -0.2211 | -0.3388 | -0.3387 | -0.6019 | -0.7532 | -0.7951 |
|  | Vol 3 | -0.0008 | -0.0321 | -0.0933 | -0.1270 | -0.2600 | -0.3749 | -0.5875 | -0.6233 | -0.5754 |
|  | Vol 4 | -0.0742 | -0.1353 | -0.1810 | -0.3801 | -0.4347 | -0.4502 | -0.6638 | -0.8095 | -1.0439 |
|  | Vol 5 | -0.1652 | -0.2726 | -0.5019 | -0.6629 | -0.7692 | -0.9845 | -1.1081 | -1.3934 | -1.4584 |
|  | Vol 6 | -0.0719 | -0.0719 | -0.0114 | -0.0114 | -0.1818 | -0.3217 | -0.4531 | -0.4266 | -0.5137 |
